# Functional Characterization of Double-Bond Reductases in Dihydro-β-Ionone Biosynthesis in *Cymbidium sinense*

**DOI:** 10.3390/plants14243804

**Published:** 2025-12-13

**Authors:** Xueqian Gao, Xinyue Li, Yunpeng Jia, Meimei Huang, Yuechong Yue, Lan Wang, Yanping Fan, Yunyi Yu

**Affiliations:** 1 College of Horticulture, South China Agricultural University, Guangzhou 510642, China; gaoxueqian2002@163.com (X.G.); xylee@scau.edu.cn (X.L.); jiayunpeng@stu.scau.edu.cn (Y.J.); 20233154023@stu.scau.edu.cn (M.H.); ycyue@scau.edu.cn (Y.Y.); wanglan@stu.scau.edu.cn (L.W.); 2 The Research Center for Ornamental Plants, South China Agricultural University, Guangzhou 510642, China

**Keywords:** *Cymbidium sinense*, floral fragrance, dihydro-β-ionone, double-bond reductase, functional characterization, metabolic engineering

## Abstract

*Cymbidium sinense* is a highly valued ornamental orchid renowned for its strong floral fragrance. In this study, dihydro-β-ionone was identified as a major volatile compound in *C. sinense* ‘Qi Hei’. Its emission increased progressively during flower development and was predominantly released from the sepals and petals. Transcriptome analysis of flowers at three developmental stages led to the identification of four double-bond reductase genes, designated *CsDBR1–CsDBR4*. Spatiotemporal expression profiling demonstrated that transcript levels of *CsDBRs* were highest in sepals and petals, showing a significant positive correlation with dihydro-β-ionone accumulation (*p* < 0.05). Heterologous expression in *Escherichia coli* and subsequent in vitro enzymatic assays confirmed that recombinant CsDBR1, CsDBR2, and CsDBR4 proteins catalyze the conversion of β-ionone to dihydro-β-ionone, whereas CsDBR3 exhibited no detectable activity. Transient expression in *Nicotiana benthamiana* leaves further verified the in planta function of *CsDBR1*, *CsDBR2*, and *CsDBR4*, resulting in elevated production of dihydro-β-ionone upon infiltration of β-ionone. Substrate specificity assays revealed that CsDBR2 and CsDBR4 also reduced 1-octen-3-one, 3-nonen-2-one, and pulegone. Collectively, these findings demonstrate that CsDBR1, CsDBR2, and CsDBR4 are key enzymes responsible for dihydro-β-ionone biosynthesis in *C. sinense*, providing a genetic foundation for molecular breeding aimed at improving floral fragrance in orchids.

## 1. Introduction

As a traditional ornamental plant in China, *Cymbidium sinense* is widely prized for its elegant floral morphology, distinctive leaf shape, and particularly its captivating fragrance. Previous research has extensively explored various aspects of this species, including flowering regulation [1], floral development [2,3,4,5], leaf coloration [6,7], disease resistance [8,9], and stress adaptation [10,11]. However, the molecular basis of its floral scent production remains less understood compared to other ornamental traits. Recent advances provide crucial foundations for further investigation: The chromosome-level genome reveals terpenoid-related biosynthetic gene clusters [1], and phytochemical analyses identify β-ionone and dihydro-β-ionone as dominant volatiles [12]. These findings establish important genomic and biochemical frameworks for elucidating scent biosynthesis in this species.

Dihydro-β-ionone serves as a principal aromatic component in *C. sinense* essential oil, distinguished by its characteristic woody-fruity aroma [12], and functions as a key fragrance compound in various plant species, including *Osmanthus fragrans* [13], *Crocus sativus* [14], *Docynia delavayi* [15], and *Taccarum ulei* [16]. Its unique organoleptic properties underpin broad applications as a flavor and fragrance additive in food, cosmetic, and detergent industries, with global annual consumption estimated at 10–100 metric tons [17]. Biochemically, it acts as a precursor for theaspirane synthesis in tobacco products and exhibits significant biological activities, including allelopathic effects inhibiting growth in *Lactuca sativa* and *Lepidium sativum* [18], suggesting potential herbicidal applications, along with insect attractant characteristics notably for *Phyllotreta cruciferae* [16,19], indicating utility in integrated pest management.

While chemical synthesis remains the primary method for industrial-scale dihydro-β-ionone production, consumer preference for plant-derived natural compounds has significantly reduced the market value of synthetic versions compared to their natural counterparts. The limited availability and high extraction costs of plant-based dihydro-β-ionone have prompted the exploration of biotechnological alternatives, with metabolic engineering emerging as a particularly promising sustainable approach [20]. For instance, a one-pot biosynthetic system converts β-apo-8′-carotenal to dihydro-β-ionone through two enzymatic steps: oxidative cleavage by *Petunia hybrida* carotenoid cleavage dioxygenase (PhCCD1) producing β-ionone, followed by reduction via *Artemisia annua* double bond reductase (AaDBR1) [21]. Despite these advances, the complete biosynthetic pathway in plants requires further elucidation, and comprehensive identification of the involved genes demands additional research.

Plant double-bond reductases (DBRs), members of the medium-chain dehydrogenase/reductase (MDR) superfamily, possess conserved structural domains that enable NADPH-dependent reduction of C=C bonds in α,β-unsaturated carbonyl compounds [22,23]. These enzymes feature a C-terminal Rossmann fold domain with parallel β-sheets and α-helix pairs arranged in β-α-β-α-β topology for cofactor binding, and an N-terminal substrate-binding domain with antiparallel β-strands and surface α-helices [24,25], forming a catalytic cleft that accommodates both cofactors and substrates. Although primarily functioning as homodimers, certain MDR members adopt monomeric or tetrameric configurations to optimize catalytic activity. Functional studies demonstrate the catalytic versatility of DBR enzymes across plant species: PtPPDBR from *Pinus taeda* reduces dehydrodiconiferyl aldehyde and coniferyl aldehyde to dihydro derivatives [22]; RZS1 from *Rubus idaeus* converts 4-hydroxybenzalacetone derivatives to raspberry ketone and gingerone [23]; multiple PfDBR isozymes in *Perilla frutescens* catalyze isoegoma ketone to perilla ketone conversion [26]; and MdHCDBR in *Malus × domestica* redirects phenylpropanoid flux toward dihydrochalcone biosynthesis via p-coumaroyl-CoA reduction [27]. These established functional roles provide compelling evidence supporting DBR involvement in dihydro-β-ionone production in *C. sinense*.

This study aims to systematically investigate the molecular basis of dihydro-β-ionone biosynthesis in *C. sinense* through an integrated approach combining volatile compound profiling, transcriptome analysis, and functional characterization of candidate genes. We first analyzed the composition and emission patterns of floral volatiles across different developmental stages and tissue types to establish the significance of dihydro-β-ionone as a key aroma component. Using transcriptome sequencing of flowers at three developmental phases, we identified candidate double-bond reductase genes (*CsDBRs*) potentially involved in the biosynthetic pathway. Through comprehensive bioinformatic analysis, heterologous expression, and enzymatic assays, we functionally characterized these CsDBRs to determine their specific roles in catalyzing the conversion of β-ionone to dihydro-β-ionone. Furthermore, we employed transient expression systems to validate their activity in planta and investigated their substrate specificity to understand their functional diversity. The findings from this research will provide valuable genetic resources and fundamental insights for molecular breeding programs aimed at floral fragrance improvement in orchids and advance our understanding of apocarotenoid metabolism in plants.

## 2. Results

### 2.1. Characterization of Volatile Compounds

**Figure 1:**
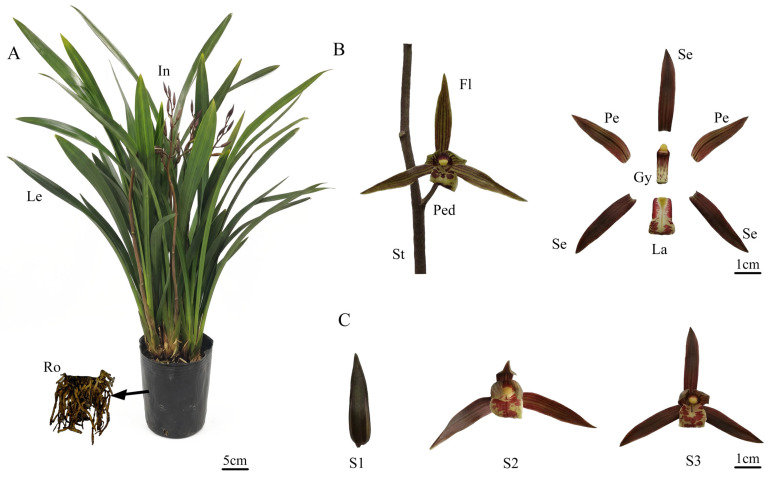
Plant morphology and floral development of *Cymbidium sinense* ‘Qi Hei’. (**A**) Schematic diagram of the whole plant architecture. Ro, root; Le, leaf; In, inflorescence. (**B**) Dissection of inflorescence and floral organs. St, stem; Ped, pedicel; Fl, Flower; Se, sepal; Pe, petal; La, labellum; Gy, gynandrium. (**C**) Three key developmental stages of the flower. S1, bud stage; S2, partial-bloom stage; S3, full-bloom stage.

The *C. sinense* plant was divided into roots, leaves, and inflorescences (Figure 1A). The inflorescences consisted of stems and flowers, which were further categorized into pedicels, sepals, petals, labellum, and gynandrium (Figure 1B). Floral development was categorized into three unique phases: the bud stage (S1), partial-bloom stage (S2), and full-bloom stage (S3) (Figure 1C). A systematic investigation of volatile organic compounds (VOCs) was undertaken in *C. sinense* ‘Qi Hei’ spanning three developmental stages (S1–S3) and four floral parts (sepals, petals, labellum, and gynandrium) using the combined technique of headspace solid-phase microextraction coupled with gas chromatography-mass spectrometry (HS-SPME-GC-MS). Table 1 shows the whole dataset of detected VOCs and their relative abundances.

Our systematic analysis of floral VOCs in *C. sinense* ‘Qi Hei’ identified nine major components, comprising five apocarotenoids and four sesquiterpenoids. β-ionone and dihydro-β-ionone were recognized as the predominant aroma compounds, collectively accounting for 92.77% of the total emissions (Table 1). The developmental dynamics revealed a significant increase in the concentrations of these key apocarotenoids throughout flower development, peaking at the full-bloom stage (S3). Specifically, dihydro-β-ionone content increased approximately 6.7-fold from the bud stage (S1: 0.16 ± 0.02%) to the full-bloom stage (S3: 1.07 ± 0.15%), while β-ionone showed an even more dramatic 9.1-fold increase (S1: 0.71 ± 0.15% to S3: 6.48 ± 0.03%). Notably, other compounds, such as farnesol, (*E*)-β-ionone, and α-ionone, exhibited distinct temporal accumulation patterns, with sesquiterpenoids specifically emerging at S3 alongside the peak production of apocarotenoids.

Tissue-specific analysis at the full-bloom stage (S3) demonstrated that sepals and petals were the primary emission sites, contributing 82.55% of the total volatile output. All floral organs contained the three key apocarotenoids—(*E*)-β-ionone, β-ionone, and dihydro-β-ionone—but completely lacked sesquiterpenoids. The spatial distribution of total volatiles, as well as the primary components (β-ionone and dihydro-β-ionone), followed a clear gradient: sepals (highest) > petals > labellum > gynandrium (lowest), confirming these floral organs as the main production and emission sites for the characteristic aroma.

### 2.2. Transcriptome Assembly and Annotation

Transcriptome sequencing was conducted on *C. sinense* flowers at three developmental stages: bud (S1), partial-bloom (S2), and full-bloom (S3), to unravel the molecular process of dihydro-β-ionone production. These stages were selected due to significant shifts in volatile compound concentrations, thereby facilitating the identification of genes associated with scent emission. Nine cDNA libraries were established, producing 63.48 Gb of raw reads. Following the removal of adaptor sequences and low-quality reads, 52.14 Gb of clean reads were acquired, with average Q20 and Q30 values above 98% and 94%, respectively, signifying good sequencing quality appropriate for subsequent analysis (Table S1). The de novo assembly yielded 177,171 transcripts and 76,703 unigenes (Table S2). The N50 lengths of transcripts and unigenes were 2276 bp and 1782 bp, with mean lengths of 1378 bp and 1112 bp, respectively, satisfying the criteria for reliable gene expression measurement and differential expression analysis (Table S3).

The unigenes were functionally annotated by comparing them to seven databases: Non-redundant protein (Nr), Non-redundant nucleotide (Nt), Protein families database (Pfam), EuKaryotic Orthologous Groups (KOG), UniProtKB/Swiss-Prot, Kyoto Encyclopedia of Genes and Genomes (KEGG), and Gene Ontology (GO). Of the 76,703 unigenes, 38,446 (50.12%) received annotation in at least one database, but only 3460 (4.51%) were annotated across all databases. The NR database exhibited the highest annotation rate, with 30,489 unigenes (39.74%), followed by the Nt database with 25,593 unigenes (33.36%). Conversely, the KOG database comprised the smallest number of annotated unigenes, totaling 5505, which represents 7.17% (Table S4).

The species distribution, *E*-value distribution, and sequence similarity distribution of the annotated unigenes were characterized based on the Nr database annotation. Most unigenes (60.4%) displayed the most significant sequence similarity to *Dendrobium catenatum*, followed by *Phalaenopsis equestris* (19.7%), *Apostasia shenzhenica* (2.8%)*, Vitis vinifera* (1.7%), and *Elaeis guineensis* (0.6%). Regarding the *E*-value distribution, 6.0% of the unigenes with significant hits demonstrated perfect matches (*E*-value = 0), 26.6% exhibited very high homology (0 < *E*-value < 1.0 × 10^−100^), and 19.8% revealed substantial homology (1.0 × 10^−100^ ≤ *E*-value < 1.0 × 10^−45^). The remaining 47.6% exhibited moderate homology (*E*-value ≥ 1.0 × 10^−45^). Moreover, the sequence similarity analysis indicated that 7.2% of unigenes exhibited 95–100% identity, 42.9% demonstrated 80–90% identity, and 37.0% displayed 60–80% identity, whereas 12.6% and 0.2% revealed 40–60% and 18–40% identity, respectively.

GO analysis classified 19,723 annotated unigenes into three functional groups. In the realm of biological processes, the predominant unigenes were linked to cellular processes, metabolic activities, biological regulation, and the regulation of biological processes. The majority were associated with binding, catalytic activity, and transporter activity in terms of molecular function. The cellular component group comprised the smallest number of unigenes, predominantly allocated to cellular anatomical entity, intracellular, and protein-containing complex (Figure S1).

According to KEGG Orthology (KO) annotations, unigenes were assigned to 368 metabolic pathways. Among these, 126 pathways were associated with metabolism, 86 with organismal systems, 32 with cellular processes, 24 with environmental information processing, and 24 with genetic information processing. Several pathways potentially implicated in apocarotenoid biosynthesis were identified, including pyruvate metabolism, carotenoid biosynthesis, zeatin biosynthesis, sesquiterpenoid and triterpenoid biosynthesis, terpenoid backbone biosynthesis, diterpenoid biosynthesis, and monoterpenoid biosynthesis (Figure S2). As an outcome, genes linked to these pathways have been selected for further investigation.

Differential gene expression analysis was performed using DESeq2 v1.26.0, applying a threshold of adjusted *p*-value (padj) ≤ 0.05 and |log_2_FoldChange| ≥ 1.0 [28]. Pairwise comparisons among the three developmental phases (S1, S2, and S3) of *C. sinense* flowers revealed a total of 10,425 differentially expressed genes (DEGs). The comparison between S1 and S3 revealed the greatest quantity of DEGs at 7143 (3826 up-regulated and 3317 down-regulated), followed by S2 vs. S3 with 6723 (3297 up-regulated and 3426 down-regulated), and S1 versus S2 with 3321 (2086 up-regulated and 1235 down-regulated) (Figure S3).

The DEGs were enriched in 240 KEGG pathways. The pathways exhibiting the highest gene enrichment encompassed phenylpropanoid biosynthesis, MAPK signaling pathway in plants, plant-pathogen interactions, flavonoid biosynthesis, plant hormone signal transduction, alpha-linolenic acid metabolism, secondary metabolite biosynthesis, photosynthesis, carotenoid biosynthesis, and starch and sucrose metabolism. This enrichment pattern establishes a basis for future studies on volatile chemical metabolism in *C. sinense* (Figure S4).

### 2.3. Identification of Transcription Factors

Transcription factors (TFs) in higher plants are crucial for numerous physiological processes, including plant growth, development, and morphogenesis, as well as the synthesis of secondary metabolites and responses to stimuli. To identify TFs in *C. sinense*, we utilized the PlantTFDB database. This analysis classified a total of 1451 genes as transcription factors, based on established criteria [29,30,31]. Among these, 613 were identified as DEGs. The DEGs were assigned to 71 transcription factor families, of which five were predominantly enriched: AP2/ERF (60 genes), MYB (45), C2H2 (39), bHLH (34), and WRKY (27). These families of transcription factors have been documented to govern the biosynthesis of carotenoids, apocarotenoids, terpenoids, and various other metabolites in plants [32,33,34,35,36]. These transcription factors were highly expressed during the S1–S3 phase. We focused on genes having expression patterns that match the release pattern of volatile compounds in *C. sinense*, indicating their possible role in aroma compound synthesis (Figure S5).

### 2.4. Analysis of Carotenoid Biometabolic Pathway

**Figure 2:**
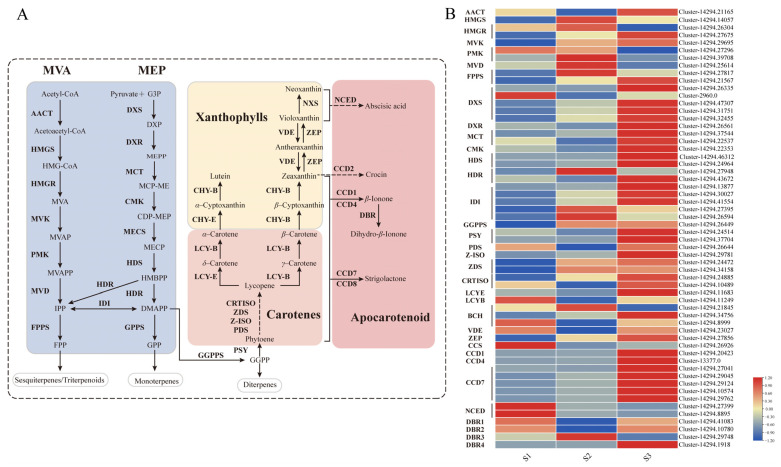
Carotenoid metabolic pathway and expression profiles of related genes in *Cymbidium sinense*. (**A**) Schematic diagram of the carotenoid biosynthetic pathway. Key intermediates and enzymes are indicated. The full names of all abbreviations are listed in the Abbreviations section. (**B**) Heatmap showing the expression levels of genes involved in carotenoid biosynthesis across three developmental stages (S1–S3).

Dihydro-β-ionone, a volatile apocarotenoid derived from carotenoid cleavage, is biosynthesized via the terpenoid pathway. In higher plants, the primary precursor for carotenoids is geranylgeranyl pyrophosphate (GGPP), which is produced through the methylerythritol phosphate (MEP) pathway. GGPP is subsequently converted into various carotenoid compounds through a series of enzymatic reactions (Figure 2A).

To investigate the transcriptional regulation of this pathway during floral development, we conducted transcriptome profiling. We identified a total of 32 genes involved in terpenoid backbone biosynthesis and 36 genes directly associated with carotenoid biosynthesis. As shown in Figure 2B, the expression levels of key genes responsible for GGPP synthesis (including *DXS*, *DXR*, *MCT*, *CMK*, *HDS*, *HDR*, *IDI*, and *GGPPS*) and for carotenoid biosynthesis (including *PSY*, *PDS*, *Z-ISO*, *ZDS*, *CRTISO*, *BCH*, and *ZEP*) all peaked sharply during the S3 developmental stage. This coordinated transcriptional peak correlates directly with the peak in volatile emission in *C. sinense*, suggesting a strong regulatory link between gene expression and metabolite production.

**Figure 3:**
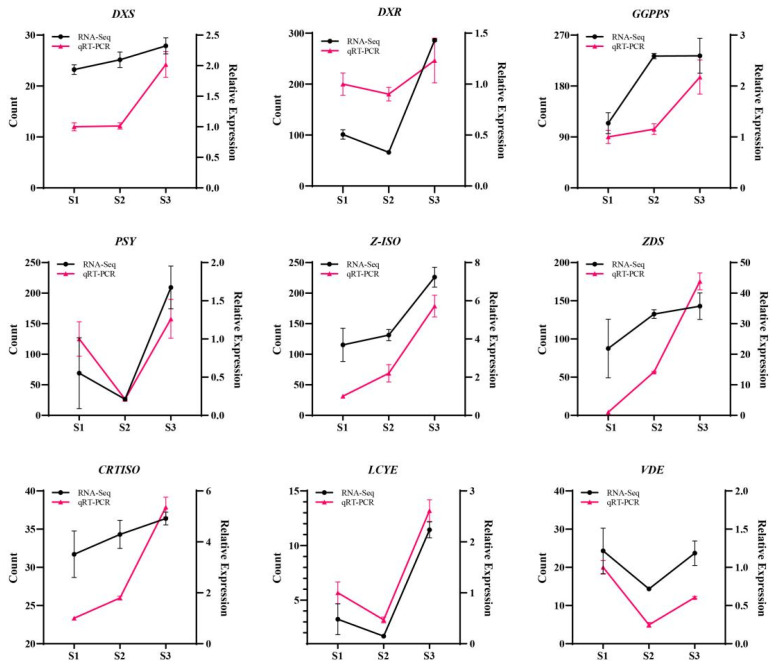
Expression profiling of key genes in the MEP pathway in *Cymbidium sinense*. *DXS*, Cluster-14294.32455; *DXR*, Cluster-14294.26561; *GGPPS*, Cluster-14294.26449; *PSY*, Cluster-14294.24514; *Z-ISO*, Cluster-14294.29781; *ZDS*, Cluster-14294.24472; *CRTISO*: Cluster-14294.24885; *LCYE*: Cluster-14294.11683; *VDE*: Cluster-14294.23027.

To validate the transcriptome data, we selected nine critical genes for qRT-PCR analysis: *DXS*, *DXR*, *GGPPS*, *PSY*, *Z-ISO*, *ZDS*, *CRTISO*, *LCYE*, and *VDE*. The qRT-PCR results confirmed the expression patterns observed in the transcriptome data. As presented in Figure 3, all nine genes showed congruent expression trends between the two independent methods, thereby robustly verifying the reliability of our transcriptomic dataset.

### 2.5. Identification, Phylogenetic and Bioinformatic Characterization of CsDBRs

The formation of dihydro-β-ionone in *C. sinense* has not been documented in the literature. Structural research indicates that DBR enzymes, similar to those identified in *Murraya* species, may facilitate the transformation of β-ionone into dihydro-β-ionone. Four potential *DBR* genes in *C. sinense*, labeled *CsDBR1* (Cluster-14294.41083), *CsDBR2* (Cluster-14294.10780), *CsDBR3* (Cluster-14294.29748), and *CsDBR4* (Cluster-14294.1918), were found using extensive transcriptome analysis utilizing TBtools v2.065 software [37]. The maximum likelihood phylogenetic analysis of DBR proteins (Figure S6) indicated substantial evolutionary divergence, with CsDBR1 exhibiting the closest relationship to CjDBR, CsDBR2 to PeDBR, and CsDBR4 to ZmDBR. The research identified two primary clades: the first comprised CsDBR2, CsDBR4, ZmDBR, PeDBR, ZoDBR, MaDBR, and CaDBR1, while the second included CsDBR3, DcDBR, and DzDBR.

SWISS-MODEL predicted the tertiary structure of CsDBR proteins and found RiZS1 from *Rubus idaeus* to be the best template for CsDBR1 (43.79% sequence identity, GMQE = 0.81) and CsDBR3 (61.93% sequence identity, GMQE = 0.87). *Zingiber officinale*’s double bond reductase (ZoDBR) has the most structural similarity to CsDBR2 and CsDBR4, with sequence identities of 73.04% (GMQE = 0.86) and 65.59%, respectively. RiZS1 and ZoDBR, previously described by X-ray crystallography, are established members of the MDR superfamily [38,39,40].

**Figure 4:**
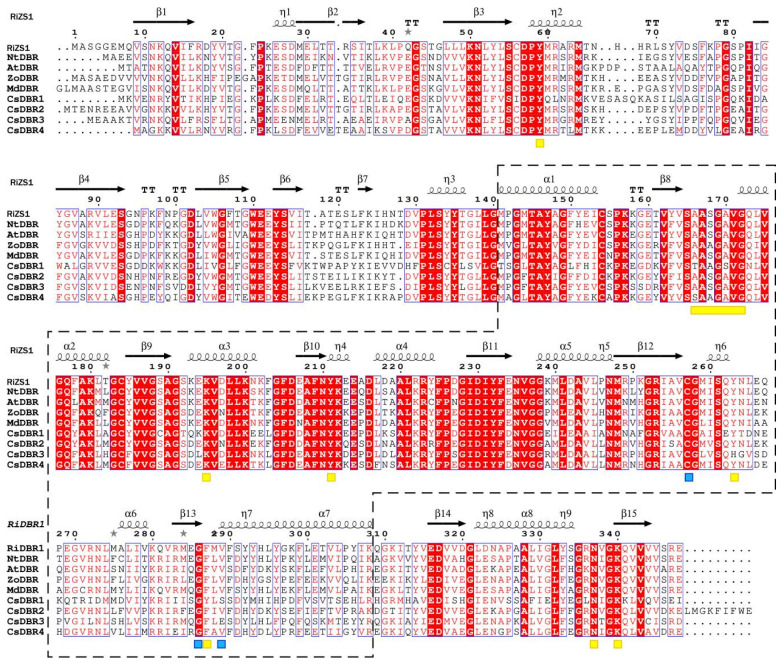
Multiple sequence alignment of CsDBRs and homologous double-bond reductases. Alignment was performed with CLUSTALW and visualized by ESPript 3.0. Secondary structure elements (α-helices, β-strands, turns) are shown above the sequences. Red background with white text indicates identical residues (complete conservation); white background with red text indicates regions of high similarity where residues are non-identical but share similar physicochemical properties. Blue boxes enclose highly conserved fragments. Arrows and "springs" denote β-sheets and α-helices, respectively; gray asterisks mark residues with alternative conformations. Nucleotide-binding domains are boxed with dashed lines. Yellow rectangles highlight NAD(P)H-binding domains; yellow squares mark key residues interacting with NAD(P)H phosphate, ribose, and nicotinamide moieties. Blue squares indicate substrate-binding residues. The dots represent amino acid sites, corresponding to the numbers above. Accession numbers: RiZS1 (*Rubus idaeus*, AEL78825.1); NtDBR (*Nicotiana tabacum*, NP_001313179.1); AtDBR (*Arabidopsis thaliana*, AAY25470.1); ZoDBR (*Zingiber officinale*, PKU75477.1); MdDBR (*Malus domestica*, 6YTZ_A).

Multiple sequence alignment with RiZS1 as a structural template revealed that CsDBR1, CsDBR2, CsDBR3, and CsDBR4 possess analogous enzyme active site structures to those of RiZS1, NtDBR, AtDBR, ZoDBR, and MdDBR (Figure 4). Structural study indicated that CsDBR proteins possess two distinctive MDR superfamily domains: a substrate-binding domain (residues 1–140 and 309–357) and a nucleotide-binding Rossmann fold (residues 141–308). The nucleotide-binding domain consists of seven α-helices (αA–αG) and six β-strands (βA–βF) organized in alternate rows, forming a characteristic parallel β-sheet shape. All four CsDBRs possess the conserved NAD(P)H-binding motif AXXGXXG characteristic of the MDR family. However, Ala166 is substituted by Thr in CsDBR1 and Ser in CsDBR4, while it remains conserved in CsDBR2 and CsDBR3. The functional consequences of these changes on cofactor binding affinity necessitate additional examination. The essential residues participating in hydrogen bonding with the nicotinamide ring (Cys257, Phe286, and Val289) are preserved in CsDBR2 and CsDBR4, mirroring the arrangement found in MdDBR. Further structural variations encompass the substitution of Phe290 with Ser290 in CsDBR1 and CsDBR3, as well as the replacement of Leu288 with Ile288 in CsDBR2 and Ala288 in CsDBR4; these modifications have been documented to induce steric hindrance, potentially affecting ligand orientation within the active site [39]. Tyr263, a catalytically important residue found in CsDBR1, CsDBR2, and CsDBR4 that enables substrate binding and hydride transfer, is replaced by His263 in CsDBR3, a change that may impact enzymatic function [41]. Comparative sequencing analysis revealed that nucleotide-binding domains are significantly more conserved than substrate-binding domains in these enzymes.

### 2.6. Spatiotemporal Expression Analysis of CsDBRs

**Figure 5:**
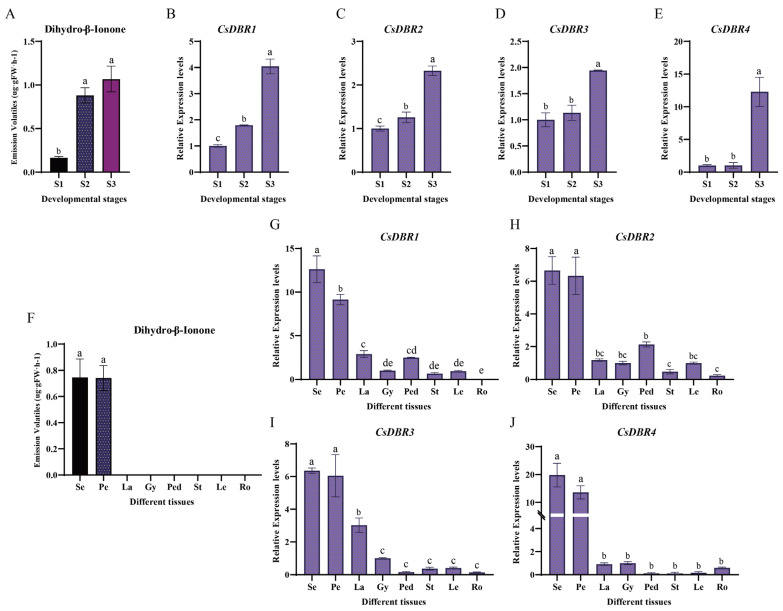
Dihydro-β-ionone emission and *CsDBRs* expression patterns during floral development and across tissues. (**A**) Dihydro-β-ionone content at three floral developmental stages (S1, bud stage; S2, partial-bloom stage; S3, full-bloom stage). (**B**–**E**) Relative expression levels of *CsDBR1* (**B**), *CsDBR2* (**C**), *CsDBR3* (**D**), and *CsDBR4* (**E**) genes during floral development. (**F**) Dihydro-β-ionone content in eight plant tissues (Se, sepal; Pe, petal; La, labellum; Gy, gynandrium; Ped, pedicel; St, stem; Le, leaf; Ro, root). (**G**–**J**) Relative expression levels of *CsDBR1* (**G**), *CsDBR2* (**H**), *CsDBR3* (**I**), and *CsDBR4* (**J**) genes across different tissues. Different letters above bars (*a–e*) indicate significant differences (one-way ANOVA with Tukey’s HSD test, *p* < 0.05). Values are presented as mean ± SD (*n* = 3).

To investigate the roles of *CsDBR* genes in dihydro-β-ionone biosynthesis, their expression profiles were analyzed across different floral developmental stages and tissues of *C. sinense*. During flower development (S1 to S3), the expression of all four *CsDBR* genes increased markedly, a pattern that closely paralleled the accumulation of dihydro-β-ionone (Figure 5A–E), indicating a developmentally regulated expression associated with scent production.

Spatially, high expression of all *CsDBR* genes was predominantly localized to the sepals and petals, the exclusive sites of dihydro-β-ionone emission (Figure 5F–J). In contrast, their expression in other tissues (e.g., labellum, gynandrium, pedicel, vegetative organs) was minimal or undetectable. This strict tissue-specific expression pattern aligns precisely with the organ-specific emission of the scent, supporting the local function of *CsDBRs* in scent synthesis.

Furthermore, statistical analysis revealed significant positive correlations (*p* < 0.05) between the expression level of each *CsDBR* gne and the quantity of dihydro-β-ionone across samples (Figure S7). Collectively, the synchronized spatiotemporal expression and strong correlation with metabolite abundance provide compelling evidence that these *CsDBR* genes are key genetic determinants for dihydro-β-ionone biosynthesis in *C. sinense*.

### 2.7. Cytoplasmic Localization of CsDBR Proteins

To determine the subcellular localization of the four candidate CsDBR proteins, bioinformatic predictions were first performed using CELLO v.2.5 and WoLF PSORT (https://wolfpsort.hgc.jp/, accessed on 27 February 2024) [42,43], which consistently indicated cytoplasmic localization for all four proteins.

**Figure 6:**
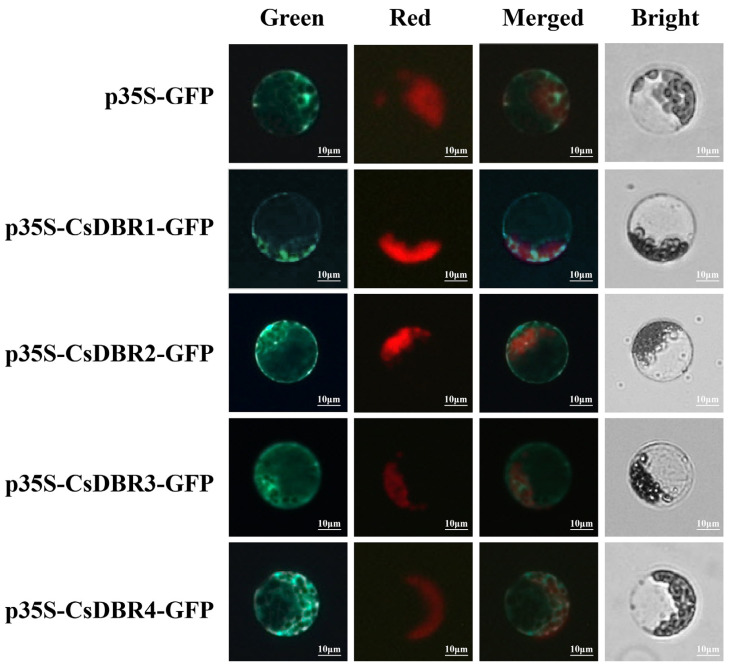
Subcellular localization of CsDBRs in *Arabidopsis* protoplasts. Fluorescence channels: Green (GFP signal); Red (Chlorophyll autofluorescence); Merged (Composite of green and red channels); Bright (Bright-field image). Scale bar = 10 μm.

For experimental validation, the coding sequences (CDSs) of each *CsDBR* were fused to the N-terminus of Green fluorescent protein (GFP) under the control of the CaMV 35S promoter, generating CsDBR-GFP fusion constructs. These were transiently expressed in *Arabidopsis* mesophyll protoplasts. As shown in Figure 6, fluorescence microscopy revealed that all four fusion proteins exhibited a diffuse green fluorescence exclusively in the cytoplasm, clearly outlining the chloroplast periphery. This pattern was distinct from chloroplast autofluorescence, confirming cytoplasmic localization. No signal was detected in the nucleus, vacuole, or other organelles.

The results provide direct visual evidence that CsDBR1–CsDBR4 are cytoplasmic proteins (Figure 6), consistent with the bioinformatic predictions. This localization is functionally relevant, as it positions these enzymes in the same compartment where their substrate β-ionone is likely produced, thereby facilitating efficient metabolic channeling toward dihydro-β-ionone biosynthesis.

### 2.8. Heterologous Expression and In Vitro Enzymatic Assays of CsDBRs

The CDSs of *CsDBR1*–*CsDBR4* were PCR-amplified (Figure S8) and cloned into the pET-32a vector (Novagen, Darmstadt, Germany) for heterologous expression in *E. coli* Rosetta (DE3). Following Ni-NTA affinity purification, SDS-PAGE analysis confirmed the successful isolation of recombinant proteins. As shown in Figure S9, all four purified fusion proteins exhibited an approximate molecular weight of 56 kDa, consistent with the expected sizes derived from the vector tag (~18 kDa) and the predicted molecular weights of each CsDBR (CsDBR1: 38.50 kDa, CsDBR2: 40.15 kDa, CsDBR3: 38.32 kDa, CsDBR4: 38.07 kDa).

**Figure 7:**
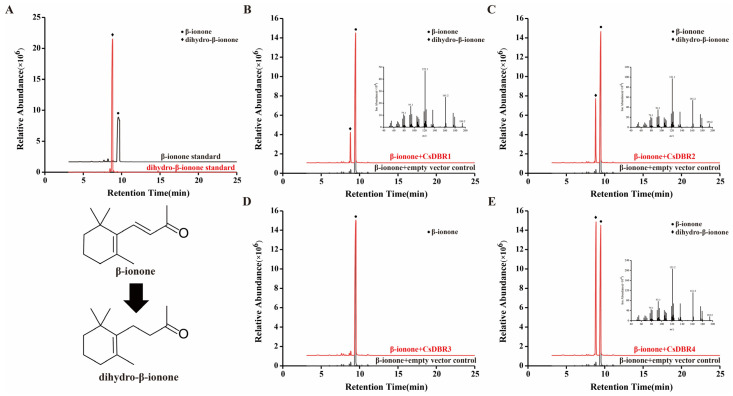
In vitro enzyme assays of recombinant CsDBR proteins showed the conversion of β-ionone to dihydro-β-ionone. GC-MS chromatograms: (**A**) β-ionone and dihydro-β-ionone standards; (**B**) CsDBR1 reaction; (**C**) CsDBR2 reaction; (**D**) CsDBR3 reaction; (**E**) CsDBR4 reaction. Circle and rhombus represent β-ionone and dihydro-β-ionone.

Subsequently, the enzymatic activities of the purified proteins were assessed through in vitro assays using β-ionone as the substrate. Gas chromatography-mass spectrometry (GC-MS) analysis revealed distinct catalytic functions. As shown in Figure 7, reactions with CsDBR1, CsDBR2, or CsDBR4 each produced a new peak corresponding to dihydro-β-ionone, confirmed by matching retention time and mass spectrum with an authentic standard. In contrast, the CsDBR3 reaction chromatogram was indistinguishable from the negative control, showing only the substrate peak. These results provide direct biochemical evidence that CsDBR1, CsDBR2, and CsDBR4 catalyze β-ionone reduction to dihydro-β-ionone, while CsDBR3 shows no detectable activity under these conditions.

To verify the catalytic activity of CsDBRs in a plant cellular context, we employed a transient expression system in *N*. *benthamiana* leaves. The CDSs of *CsDBR1*, *CsDBR2*, and *CsDBR4* were cloned into the pGreenII 62-SK vector. The recombinant plasmids were introduced into *Agrobacterium tumefaciens* strain GV3101 (pSoup) and infiltrated into leaves of *N. benthamiana* plants. Control leaves were infiltrated with the empty vector. Three days post-infiltration, the leaf tissues were injected with β-ionone (3 mM) to provide substrate for the transiently expressed enzymes.

**Figure 8:**
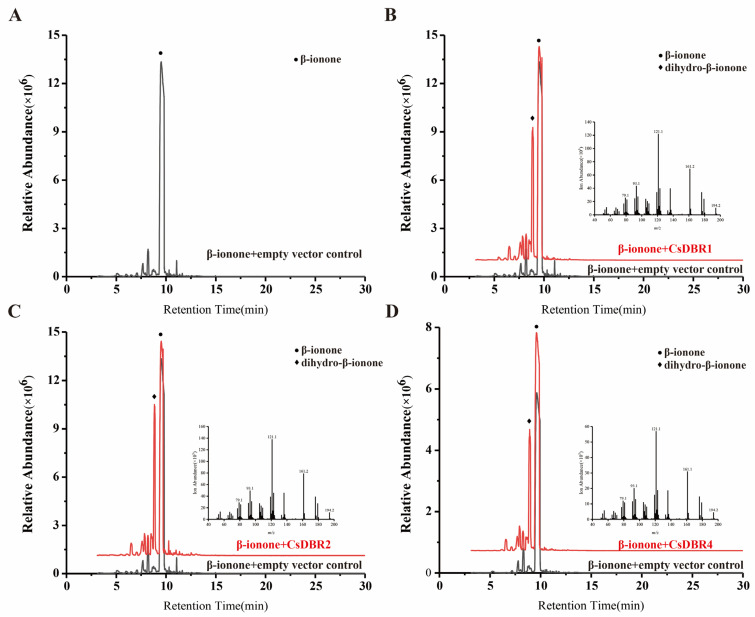
In vivo conversion of β-ionone to dihydro-β-ionone by transiently expressed *CsDBRs* in *Nicotiana benthamiana* leaves. (**A**) Chromatogram of pGreenII 62-SK empty vector; (**B**) Chromatogram and Mass Spectrum of CsDBR1; (**C**) Chromatogram and Mass Spectrum of CsDBR2; (**D**) Chromatogram and Mass Spectrum of CsDBR4. Circle and rhombus represent β-ionone and dihydro-β-ionone.

Volatile compounds were analyzed using HS-SPME-GC-MS 24 h after β-ionone injection. As shown in Figure 8, GC-MS analysis revealed a distinct new peak in leaves expressing *CsDBR1*, *CsDBR2*, or *CsDBR4*, which was absent in control leaves. This compound was identified as dihydro-β-ionone based on its retention time and mass spectrum matching an authentic standard. The mass spectrum showed characteristic fragment ions, including the molecular ion [M]^+^ at *m*/*z* 194 and the base peak at *m*/*z* 137, consistent with dihydro-β-ionone. In contrast, control samples exhibited only the β-ionone substrate peak. These results provide direct evidence that CsDBR1, CsDBR2, and CsDBR4 catalyze the conversion of β-ionone to dihydro-β-ionone in planta, confirming their functional role in this biosynthetic pathway.

To explore the substrate specificity of these four proteins, 1-octen-3-one, 3-nonen-2-one, (*R*)-(+)-pulegone, and α-methylcinnamaldehyde, having a double-bond structure similar to β-ionone, were employed as substrates for in vitro enzyme activity experiments. GC-MS analyses demonstrated that CsDBR1, CsDBR2, CsDBR3, and CsDBR4 are capable of catalyzing the transformation of 1-octen-3-one into 3-octanone; however, CsDBR3 had diminished activity, yielding only negligible quantities of 3-octanone. When 3-nonen-2-one served as the substrate, CsDBR1, CsDBR2, and CsDBR4 yielded 2-nonanone. When (*R*)-(+)-pulegone served as the substrate, CsDBR2 and CsDBR4 facilitated the synthesis of menthone and isomenthone, while CsDBR1 and CsDBR3 exhibited no reducing activity. Likewise, GC-MS analysis of the reaction with α-methylcinnamaldehyde indicated that none of the enzymes yielded 2-methyl-3-phenylacetone. In vitro enzymatic studies demonstrated that CsDBR1, CsDBR2, and CsDBR4 possess unique substrate selectivities, with CsDBR2 and CsDBR4 displaying a wider substrate specificity among the evaluated compounds. Conversely, CsDBR3 exhibited virtually no reductase activity toward most substrates, with negligible catalytic capability except for trace conversion of 1-octen-3-one (Figures S10–S13).

## 3. Discussion

The Orchidaceae represents one of the most diverse families of angiosperms, comprising five subfamilies, more than 750 genera, and approximately 28,000 species with a nearly cosmopolitan distribution (excluding Antarctica) [44]. This plant family is characterized by three defining traits: distinctive flower morphology, varied coloration patterns, and intricate fragrance profiles. Floral smell functions as a significant decorative characteristic in orchids and is essential for pollinator attraction, stress resilience (both biotic and abiotic), and inter-plant interactions. Aromatic orchid species, owing to their multifunctional features, have become highly valued commodities in the global horticultural industry, greatly shaping consumer tastes and commanding considerable financial worth [45].

### 3.1. Analysis of Floral Fragrance Compounds and Release Patterns

Studies on floral scent components and their release patterns have revealed specific profiles in various orchids. In *Cymbidium goeringii*, the aroma is primarily composed of farnesol, methyl jasmonate (MeJA), (*E*)-β-farnesene, and nerolidol, with farnesol release peaking on the second day of anthesis [46]. In *C. sinense*, fifty-eight VOCs were identified. The emission of key monoterpenoids and sesquiterpenoids—including farnesol, (*E*)-β-farnesene, α-farnesene, and β-bisabolene—significantly increased at full-bloom compared to the bud stage [1], corroborating the general trend of rising volatile emissions during floral development observed in *C. goeringii*, *Rosa Damascena*, and *Dianthus caryophyllus* [46,47,48].

Furthermore, tissue-specific emission patterns have been documented. In *C. ensifolium*, MeJA was predominantly released from the sepals and petals, with minimal emission from the column and labellum. Similarly, in the hybrid *Cymbidium* ‘Sunny Bell’ (*C. karan* × *C. eburneum*), the sepals and petals emitted monoterpenoids, while the labellum and column released fatty acid derivatives [49]. This pattern is also evident in *C. sinense* ‘Qi Hei’, where the sepals and petals were the principal emission sites for volatiles like β-ionone and dihydro-β-ionone, with no detectable emission from the column. The expression hotspots of *CsDBRs* in these tissues further support this tissue-specific release pattern (Figure 5).

### 3.2. Functional Diversification of CsDBR Enzymes in C. sinense

The combined abundance of dihydro-β-ionone, an essential aromatic molecule in *C. sinense*, and its precursor β-ionone represents 92.77% of the volatile compounds in ‘Qi Hei’ flowers. This chemical has been recognized in various plants, including *Crocus Sativus* [14], *Polygonum chinense* [18], *Prunus persica* var. *compressa* [50], *Crataegus mollis* [51], and *Hyssopus cuspidatus* [52]. β-ionone has various biological activities, including attracting pollinators, defending against biotic and abiotic stressors, and regulating plant endophytic fungus populations [53,54]. The evaluation of the dihydro-β-ionone biosynthesis pathway in *C. sinense* is crucial for the precise enhancement of orchid aroma quality.

The identification of dihydro-β-ionone as the principal volatile from *C. sinense*, which structurally differs from β-ionone by the reduction of the C7=C8 double bond, prompted the hypothesis that a double-bond reductase is involved in its biosynthesis. Prior research suggests that reductases facilitating C=C bond reduction often belong to the medium-chain dehydrogenase/reductase (MDR) superfamily, which is extensively found in microorganisms, mammals, and plants. These enzymes facilitate the production of many metabolites by reducing C=C bonds in α,β-unsaturated aldehydes, ketones, and carboxylic acids [40,55]. For instance, CaDBR1 facilitates the conversion of 4-coumaraldehyde to 4-hydroxydihydrocinnamaldehyde (4-HDCA), a crucial stage in the colchicine biosynthetic process of the medicinal plant *Colchicum autumnale* [56]. In peppermint (*Mentha* × *piperita*), MpDBR converts pulegone to menthone and isomenthone [57]. A novel monoterpene double-bond reductase, ipsdienone reductase (IDONER), part of the MDR superfamily, was found using transcriptome sequencing in the pinewood nematode (*Bursaphelenchus xylophilus*), catalyzing the conversion of ipsdienone to ipsenone [58].

Four *CsDBR* genes were discovered in *C. sinense*, all of which were bioinformatically validated as constituents of the *MDR* superfamily. Phylogenetic analysis indicated that CsDBR2, CsDBR3, and CsDBR4 clustered together, although CsDBR1 constituted a separate clade with *Cryptomeria japonica* CjDBR (Figure S6). Multiple sequence alignment with established DBRs (e.g., RiZS1, MdDBR, AtDBR) revealed approximately 70.24% sequence similarity. All four CsDBRs possess the typical NAD(P)H-binding domain (AXXGXXG); however, Ala166 is replaced by Thr in CsDBR1 and Ser in CsDBR4, a mutation that may affect coenzyme binding effectiveness. Additionally, critical conserved residues (Tyr59, Lys195, Tyr211, Asn337, Lys340) that form hydrogen bonds with the NAD(P)H cofactor were identified. A significant exception was found at position 263: whereas CsDBR1, CsDBR2, and CsDBR4 contain the conserved Tyr263, CsDBR3 has it replaced by His263. Tyr263 has a crucial role in establishing hydrogen bonds between NADP(H) and the substrate in *Arabidopsis* AtDBR, facilitating acid-base transfer during the reduction of enal substrates [41]. The Tyr263 → His263 mutation in CsDBR3 is anticipated to modify the active-site microenvironment, potentially hindering coenzyme binding or substrate orientation.

To functionally evaluate the catalytic roles of the four discovered *CsDBRs* in dihydro-β-ionone production, each gene was cloned and produced in a prokaryotic system, and the recombinant proteins were purified to homogeneity. In vitro enzyme experiments combined with GC–MS analysis showed that CsDBR1, CsDBR2, and CsDBR4 effectively accelerated the transformation of β-ionone to dihydro-β-ionone, but CsDBR3 showed no measurable activity. This functional impairment aligns with our previous bioinformatic research, which detected a Tyr263 → His263 mutation in CsDBR3. In *Arabidopsis* AtDBR, Tyr263 is crucial for enabling hydrogen bonding between NADP(H) and the substrate, as well as for promoting acid-base catalysis [41]. We propose that the Tyr-to-His substitution alters the electrostatic microenvironment of the active site, potentially impeding cofactor binding or substrate positioning. DBR enzymes from various species frequently exhibit broad substrate promiscuity. For instance, CaDBR1 from *C. autumnale* reduces double bonds in 4-hydroxycinnamaldehyde and coniferyl aldehyde [56], multiple PfDBRs from *P. frutescens* act on substrates such as pulegone and 3-nonen-2-one [26], and NtDBR from *N. tabacum* recognizes at least 13 distinct C=C-containing compounds [40].

This study indicates that CsDBR2 and CsDBR4 can reduce diverse substrates, including 1-octen-3-one, 3-nonen-2-one, and pulegone, a capability primarily determined by the architecture of their substrate-binding domains, with residues such as Cys257 and Phe286 contributing to efficiency by stabilizing the NADPH cofactor. This functional divergence is further illustrated by the Ala166→Thr alteration in CsDBR1, which did not influence catalytic activity towards 1-octen-3-one or 3-nonen-2-one, indicating that this residue is less essential than Tyr263 for coenzyme binding [41]. CsDBR3 generated only minimal amounts of 3-octanone from 1-octen-3-one, likely attributable to mutation-induced steric hindrance that restricts the accommodation of bigger substrates (e.g., cyclic β-ionone or monocyclic pulegone). Our results demonstrate the multi-substrate catalytic activity of *C. sinense* CsDBR, with functional differences indicating enzyme-substrate interaction specificity, hence offering possibilities for optimizing catalytic efficiency through protein engineering.

### 3.3. Catalytic Efficiency and Metabolic Channeling of CsDBRs in the Cytosol

To verify the catalytic function of CsDBRs in a plant cellular environment, we employed the tobacco (*N*. *benthamiana*) transient expression system, a well-established platform renowned for its simplicity, speed, and well-defined metabolic background, which facilitates the rapid functional characterization of plant genes and secondary metabolites [59]. This robust method, further optimized for efficient compound production and analysis, has been successfully applied to characterize enzymes involved in floral scent biosynthesis. A representative example is the functional characterization of HcBSMT1 and HcBSMT2, two benzoic acid/salicylic acid carboxyl methyltransferases from *Hedychium coronarium*. Their in vitro catalytic activities—methylating benzoic acid and salicylic acid to form the volatile compounds methyl benzoate and methyl salicylate—were conclusively verified in vivo via tobacco transient expression followed by substrate infiltration, which resulted in the emission of these detectable floral scent volatiles [60]. Applying this reliable approach, we demonstrated that CsDBR1, CsDBR2, and CsDBR4 catalyze the conversion of β-ionone to dihydro-β-ionone in planta (Figure 8).

The functional relevance of this catalytic activity is further underscored by the subcellular compartmentalization of the CsDBR enzymes. Our heterologous expression in *E. coli* and transient assays in *N. benthamiana* confirmed their capability to reduce β-ionone, a finding strongly supported by their definitive localization to the cytoplasm (Figure 6). Critically, as the substrate β-ionone is generated by cytosolic carotenoid cleavage dioxygenases (CCDs) [1,21], the co-localization of CsDBRs and their substrate provides a structural basis for efficient metabolic channeling, potentially enabling the immediate conversion of β-ionone upon its generation. This spatial organization ensures immediate substrate conversion, facilitating the rapid synthesis of dihydro-β-ionone and thereby explaining its synchronized emission with *CsDBR* gene expression during flower development.

While this study solidifies the catalytic and cellular basis for dihydro-β-ionone production, the in vivo regulatory mechanisms governing CsDBRs remain to be explored. Our transcriptome analysis revealed the coordinated upregulation of key carotenoid pathway genes during the full-bloom stage, suggesting a potential interplay between CsDBRs and upstream dioxygenases. Future work employing techniques such as yeast one-hybrid assays, ChIP–qPCR, or CRISPR–Cas9 gene editing will be crucial to elucidate the transcriptional regulation of *CsDBRs* by factors like MYBs and bHLHs, and to clarify their comprehensive roles within the floral fragrance network of *C. sinense*.

## 4. Materials and Methods

### 4.1. Plant Materials and Growth Conditions

The *C. sinense* ‘Qi Hei’ used in this study was sourced from Yutian Horticultural Farm in Wengyuan County, Guangdong Province, China (coordinates: 24.34° N, 114.16° E). This cultivar was selected for its high floral scent emission intensity, stable dihydro-β-ionone production across developmental stages, and representative floral morphology typical of the *C. sinense* species. It was cultivated in a greenhouse at South China Agricultural University, Guangzhou, China (23.16° N, 113.36° E). For transcriptome sequencing, samples representing three developmental stages of *C. sinense*—bud (S1), partial-bloom (S2), and full-bloom (S3) (Figure 1)—were collected between 11:00 and 13:00. Tissues from three plants per stage were pooled, flash-frozen in liquid nitrogen, and stored at –80 °C. For *Agrobacterium* infiltration assays, *N. benthamiana* was grown in a controlled chamber for approximately 50 days.

### 4.2. GC-MS Profiling of Volatile Compounds

Floral VOCs from *C. sinense* ‘Qi Hei’ were collected via headspace solid-phase microextraction (HS-SPME) and analyzed by gas chromatography-mass spectrometry (GC-MS). Fresh floral tissues (300 mg) were sealed in 20 mL headspace vials with 0.865 μg ethyl decanoate (≥99.0%, Sigma-Aldrich, Saint Louis, MO, USA; Cat. No. 22350) added as an internal standard. After equilibration at 26 °C for 1 h, volatiles were adsorbed using a PDMS/DVB/CAR fiber (Supelco, Bellefonte, PA, USA) for 1 h [46]. GC-MS was performed on an Agilent 7890B/5977B system (Agilent, Santa Clara, CA, USA) equipped with a DB-5MS column (Agilent, Santa Clara, CA, USA; 30 m × 0.25 mm × 0.25 μm) and helium as carrier gas. The oven program was: 100 °C (2 min), increased to 170 °C at 10 °C/min (2 min), then to 250 °C at 5 °C/min, and finally to 280 °C (5 min) [61]. Mass spectra were acquired in scan mode (*m*/*z* 35–500). Compounds were identified by comparing retention times and mass spectra with those in the NIST/EPA/NIH Mass Spectral Library (version 2008; National Institute of Standards and Technology, Gaithersburg, MD, USA) and authentic standards. The authentic standards, namely β-ionone and dihydro-β-ionone (purity ≥ 95%), were purchased from Tokyo Chemical Industry Co., Ltd. (TCI, Shanghai, China) and Shanghai Aladdin Biochemical Technology Co., Ltd. (Shanghai, China), respectively. Three to five biological replicates were analyzed per sample using Agilent ChemStation (version E.02.02.1431) software.

### 4.3. RNA Extraction and Transcriptome Sequencing

Approximately 100 mg of fresh plant material was subjected to total RNA extraction using the TIANGEN Polysaccharide Polyphenol RNA Kit (TIANGEN, Beijing, China). RNA integrity and concentration were assessed using an Agilent 2100 Bioanalyzer (Agilent, Santa Clara, CA, USA). Poly(A)+ mRNA was then enriched, fragmented, and used to construct cDNA libraries with the NEBNext^®^ Ultra™ RNA Library Prep Kit (New England Biolabs, Ipswich, MA, USA). Library quality was evaluated by Qubit 2.0 Fluorometer quantification, Agilent 2100 Bioanalyzer size analysis, and qPCR. Finally, three biological replicates were sequenced on an Illumina NovaSeq 6000 platform (Illumina, San Diego, CA, USA). The sequencing service was provided by Beijing Novogene Technology Co., Ltd. (Beijing, China).

Raw reads were filtered to remove low-quality, adapter-contaminated, and ambiguous sequences. Clean reads were de novo assembled using Trinity, and transcript clusters were defined with Corset and the H-Cluster algorithm to establish final gene sets. Assembly completeness was assessed with BUSCO.

Gene functions were annotated by BLAST searches (*E*-value ≤ 1 × 10^−5^) against seven databases: NR, NT, Pfam, Swiss-Prot, KOG, KEGG and GO.

Gene expression levels were quantified with Subread. The DEGs were identified using DESeq2 v1.26.0 (|log_2_FoldChange| ≥ 1, FDR ≤ 0.05). GO enrichment analysis was conducted using GOSeq (v1.32.0), while KEGG pathway enrichment was performed with KOBAS (v3.0), with a corrected *p*-value < 0.05 considered significant in both analyses.

### 4.4. Validation of Transcriptomic Data Using qPCR

To validate transcriptomic data, qRT-PCR was performed following established methodology [62]. Approximately 100 mg of plant material was used for total RNA extraction with the Plant RNA Kit (OMEGA Bio-Tek, Norcross, GA, USA), and cDNA was synthesized with the *Evo M-MLV* Reverse Transcription Kit (Accurate Biotechnology, Hunan, China). Amplification utilized the Hieff qPCR SYBR Green Master Mix (Yeasen, Shanghai, China) on an ABI 7500 Real-Time PCR System (Applied Biosystems, Carlsbad, CA, USA). The PCR reaction program was as follows: 95 °C for 5 min; followed by 40 cycles of 95 °C for 10 s, 55 °C for 20 s, and 72 °C for 30 s. Three biological and technical replicates were analyzed per sample. *ACTIN* served as the endogenous control [63], and relative expression was calculated via the 2^−ΔΔCT^ method [64]. Gene-specific primers used for qRT-PCR are listed in Table S5.

### 4.5. Identification and Structural Characterization of DBRs

The putative DBRs in *C. sinense* were identified by querying a local transcriptome database with known plant DBR amino acid sequences, using the BLAST tool in TBtools v2.065 software (*E*-value ≤ 1 × 10^−5^) [37]. Candidate CsDBR proteins were subsequently characterized through a series of in silico analyses. Their conserved domains were identified via the NCBI Conserved Domain Database, and key physicochemical parameters were calculated using Expasy ProtParam (https://web.expasy.org/protparam/, accessed on 30 November 2023). Transmembrane domains and hydrophobicity profiles were predicted with TMHMM-2.0 (https://services.healthtech.dtu.dk/services/TMHMM-2.0/, accessed on 6 December 2023) and Expasy ProtScale (https://web.expasy.org/protscale/, accessed on 6 December 2023), respectively. Secondary structure predictions were performed with SOPMA (https://npsa-prabi.ibcp.fr/cgi-bin/npsa_automat.pl?page=npsa_sopma.html, Accessed on 5 January 2024), and tertiary structural models were generated using SWISS-MODEL (https://swissmodel.expasy.org/, Accessed on 12 January 2024). All web servers were accessed through their standard online portals.

### 4.6. Phylogenetic and Structural Comparative Analysis of DBRs

A phylogenetic tree was constructed using the maximum likelihood method (bootstrap replicates = 1000) in TBtools v2.065 software, with visualization performed using the online ChiPlot tool (https://www.chiplot.online/, accessed on 2 December 2023) [65]. Multiple sequence alignment of DBR proteins from six species was performed using ClustalW (https://www.genome.jp/tools-bin/clustalw, accessed on 3 December 2023). Structural comparisons of *C*. *sinense* CsDBR proteins were performed with ESPript 3.0 (https://espript.ibcp.fr/ESPript/cgi-bin/ESPript.cgi, accessed on 5 December 2023) using the published crystal structure of *Ri*DBR1 (PDB: 6EOW) as a template.

### 4.7. Subcellular Localization Analysis

The CDSs of *CsDBRs* were cloned into a p35S-GFP vector via homologous recombination to generate GFP-fusion constructs for subcellular localization analysis, following the established strategy for *HcTPS* gene studies [66]. Isolated protoplasts from 5- to 7-week-old *Arabidopsis thaliana* rosette leaves were used for transfection. Specifically, leaf strips (0.5–1 mm) were digested in enzyme solution at 25 °C with gentle shaking (60 rpm) for 1.5–2.5 h. The reaction was terminated with 10 mL W5 solution, and the protoplasts were filtered (75-μm mesh), pelleted (100× *g*, 2 min), and resuspended in W5 solution. After a 30 min incubation on ice, protoplasts were resuspended in MMG solution to a final density of 2 × 10^5^ cells/mL. For transfection, 10–20 μg of plasmid (>1 μg/μL) was mixed with 100 μL protoplasts, followed by an equal volume of PEG solution. After 15 min at room temperature, the reaction was diluted with 2 × volume W5 solution and centrifuged (100× *g*, 2 min). The pelleted protoplasts were resuspended in 600 μL WI solution and incubated in darkness for 12–16 h before imaging GFP fluorescence under a Zeiss Axio Scope fluorescence microscope (Carl Zeiss AG, Jena, Germany).

### 4.8. Cloning and Heterologous Expression of CsDBRs in E. coli

To heterologously express *CsDBR* candidate genes in *E. coli*, their CDSs were PCR-amplified using Phanta Max Super-Fidelity DNA Polymerase (Vazyme, Nanjing, China) and cloned into the pET-32a vector via the ClonExpress II One Step Cloning Kit (Vazyme, Nanjing, China), generating recombinant constructs pET-32a-DBR1–DBR4. Primers for gene cloning and vector construction are provided in Table S5. Following transformation into *E. coli* Rosetta (DE3) competent cells, positive transformants were selected by colony PCR and grown overnight at 37 °C (180 rpm) in LB medium containing kanamycin (100 μg mL^−1^).

For protein expression, overnight cultures were diluted 1:100 into fresh LB medium. When the OD_600_ reached 0.4–0.6, expression was induced with 0.1 mM IPTG and carried out at 16 °C for 20–24 h (120 rpm). Cells were harvested by centrifugation (5000× *g*, 10 min, 4 °C), resuspended in lysis buffer (50 mM NaH_2_PO_4_, 300 mM NaCl, 10 mM imidazole, pH 8.0), and disrupted on ice by multiple rounds of sonication (30 cycles per round; 3 s pulse, 5 s interval; with 2 min cooling between rounds). After centrifugation (12,000× *g*, 20 min, 4 °C), the supernatant was subjected to purification using Ni-NTA Sefinose™ Resin 6FF (Sangon Biotch, Shanghai, China) according to the manufacturer’s protocol. Protein size was confirmed by SDS-PAGE.

### 4.9. Enzyme Activity Assay and Product Analysis

Enzyme activity assays were conducted in 200 μL reactions containing 50 mM Tris-HCl (pH 7.5), 100 μM NADPH, 20 μL purified protein, and 500 μM substrate (1-octen-3-one, 3-nonen-2-one, (*R*)-(+)-pulegone, or α-methyl-*trans*-cinnamaldehyde). After incubation at 37 °C for 1 h, volatile products were collected from the headspace using PDMS fibers (Supelco, Bellefonte, PA, USA) for 30 min and analyzed by GC-MS.

For β-ionone and α-methyl-*trans*-cinnamaldehyde, GC-MS conditions matched those used in floral volatile analysis. The conditions for 1-octen-3-one, 3-nonen-2-one, and (*R*)-(+)-pulegone were: initial oven temperature 40 °C (hold 3 min), ramped to 250 °C at 5 °C/min; electron ionization at 70 eV; transfer line 280 °C; ion source 170 °C; total run time 28 min.

### 4.10. Transient Expression of CsDBRs in N. benthamiana

For transient expression in *N. benthamiana*, the CDSs of *CsDBR1–CsDBR4* were amplified by PCR using Phanta Max Super-Fidelity DNA Polymerase and cloned into the pGreenII 62-SK vector using the ClonExpress II One Step Cloning Kit, generating the respective pGreenII 62-SK-DBR constructs. Primers for gene cloning and vector construction are provided in Table S5. These plasmids were introduced into *A*. *tumefaciens* strain GV3101 (pSoup).

Bacterial suspensions were adjusted to OD_600_ ≈ 0.4 in infiltration buffer (10 mM MES, pH 5.2, 10 mM MgCl_2_, 0.1 mM acetosyringone) and incubated for 3 h at room temperature. *N. benthamiana* leaves were then infiltrated with the suspension using needleless 1 mL syringes. After 3 days, 3 mM β-ionone was infiltrated into the same leaves. Volatiles were collected by enclosing treated leaves in a 1 L glass vial and analyzed by GC-MS under the conditions described for floral volatile analysis [61].

## 5. Conclusions

This study identified CsDBR1, CsDBR2, and CsDBR4 as the functional double-bond reductases (DBRs) responsible for the final step in dihydro-β-ionone biosynthesis in *C. sinense*. Our integrated analysis revealed that the spatiotemporal expression patterns of these three *CsDBR* genes were tightly synchronized with the emission peak of dihydro-β-ionone, particularly in the scent-producing sepals and petals during full bloom. Heterologous expression in *E. coli* and in vitro enzymatic assays confirmed that recombinant CsDBR1, CsDBR2, and CsDBR4, but not CsDBR3, catalyze the conversion of β-ionone to dihydro-β-ionone. Structural analysis suggested that the catalytic competence of the active enzymes is associated with conserved residues, including the critical Tyr263, whereas a Tyr263→His263 substitution likely underlies the inactivity of CsDBR3. Furthermore, substrate specificity assays indicated functional divergence among the active isoforms: CsDBR2 and CsDBR4 exhibited broad promiscuity towards both linear and cyclic enones, while CsDBR1 showed a narrower range. The in vivo functionality of CsDBR1, CsDBR2, and CsDBR4 was unequivocally validated through transient expression in *N. benthamiana* leaves, and their cytoplasmic localization positions them optimally to access the cytosolic precursor, β-ionone. Collectively, these findings provide a comprehensive molecular and enzymatic basis for dihydro-β-ionone formation in *C. sinense*.

The main limitations of this study lie in the unresolved upstream regulatory mechanisms. It remains unknown which transcription factors, for example, MYBs or bHLHs, control the developmental expression of the *CsDBR* genes. Moreover, the specific contribution of each active CsDBR isoform to the total production of dihydro-β-ionone in planta is not clear, as functional redundancy or isoform-specific post-translational regulation may obscure their individual roles. Additionally, although β-ionone has been confirmed as the direct substrate, its in vivo availability and the overall flux through the apocarotenoid pathway could be influenced by developmental and environmental factors not fully explored here.

Future research should address these questions through multiple approaches. Generating *CsDBR* knockout lines in *C. sinense* using CRISPR-Cas9 gene editing would clarify the functional contribution and potential redundancy of each isoform. Identifying the upstream transcription factors that regulate *CsDBR* expression also requires attention, for which techniques like yeast one-hybrid screening or ChIP-seq would be valuable. Furthermore, protein engineering strategies—including site-directed mutagenesis based on our structural insights—could be applied to improve the catalytic efficiency or tailor the specificity of these enzymes for metabolic engineering. Together, these directions will advance our understanding of apocarotenoid metabolism in orchids and facilitate the molecular breeding of ornamental plants with enhanced fragrance.

## Figures and Tables

**Table 1:** Volatile compounds of *Cymbidium sinense* ‘Qi Hei’.

ID	Name	RT	CAS	MS	Foluma	Type	Relative Content (%)						
							S1	S2	S3	Sepal	Petal	Labellum	Gynandrium
1	(*E*)-β-Ionone	7.38	79-77-6	96	C_13_H_20_O	Apocarotenoid	0.02 ± 0.01c	0.11 ± 0.04bc	0.33 ± 0.07a	0.23 ± 0.079ab	0.24 ± 0.026ab	—	—
2	Megastigma-4,6(*E*),8(*E*)-triene	7.61	51468-85-0	96	C_13_H_20_	Apocarotenoid	—	—	0.03 ± 0.001	—	—	—	—
3	α-Ionone	8.43	79-76-5	95	C_13_H_20_O	Apocarotenoid	—	0.04 ± 0.003	—	—	—	—	—
4	Dihydro-β-ionone	8.55	17283-81-7	98	C_13_H_22_O	Apocarotenoid	0.16 ± 0.02c	0.88 ± 0.09ab	1.07 ± 0.15a	0.746 ± 0.14b	0.742 ± 0.096b	—	—
5	(*E*)-β-Farnesene	8.77	18794-84-8	98	C_15_H_24_	Sesquiterpenoid	—	—	0.1 ± 0.01	—	—	—	—
6	β-Ionone	9.16	14901-07-6	98	C_13_H_20_O	Apocarotenoid	0.71 ± 0.15d	5.58 ± 0.15a	6.48 ± 0.03a	4.27 ± 0.71b	4.00 ± 0.25b	2.16 ± 0.56c	—
7	α-Farnesene	9.42	502-61-4	94	C_15_H_24_	Sesquiterpenoid	—	—	0.04 ± 0.003	—	—	—	—
8	β-Bisabolene	9.53	495-61-4	91	C_15_H_24_	Sesquiterpenoid	—	—	0.02 ± 0.001	—	—	—	—
9	Farnesol	13.3	4602-84-0	91	C_15_H_26_O	Sesquiterpenoid	—	—	0.2 ± 0.06	—	—	—	—

Note: Compound identification was based on a comparison of Retention Time (RT) and Mass Spectrum (MS) with authentic standards and the NIST library. CAS, the Chemical Abstracts Service registry number. Figures in the table are means and standard errors. a, b, c, and d within a row refer to significant differences (*p* < 0.05). Different letters indicate significant differences among means according to ANOVA analysis (*p* < 0.05). All data are presented as mean ± SD (*n* = 3). The symbol — in the table denotes that the volatile compound was not detected.

## Data Availability

All data are displayed in the manuscript and Supplementary Materials.

## Supplementary Materials

The following supporting information can be downloaded at: https://www.mdpi.com/article/10.3390/plants14243804/s1, Figure S1: Gene Ontology (GO) classification of annotated unigenes from *C. sinense* transcriptome.; Figure S2: KEGG pathway classification of annotated unigenes from *C. sinense* transcriptome.; Figure S3: Analysis of differentially expressed genes (DEGs) among three comparison groups.; Figure S4: KEGG pathway enrichment analysis of differentially expressed genes (DEGs).; Figure S5: Gene expression of transcription factors. (A) AP2/ERF, (B) MYB, (C) C2H2, (D) bHLH, (E) WRKY.; Figure S6: Comparative phylogenetic analysis of CsDBRs and other plant DBR proteins.; Figure S7: Heat map representation of correlation analysis between dihydro-β-ionone and selected *DBR* genes.; Figure S8: Agarose gel electrophoresis of *CsDBR* amplification products. M: D2000 DNA marker.; Figure S9: SDS-PAGE of the purified CsDBR enzyme.; Figure S10: GC-MS analysis of CsDBR enzymatic activity with 1-octen-3-one.; Figure S11: GC-MS analysis of CsDBR enzymatic activity with 3-nonen-2-one.; Figure S12: GC-MS analysis of CsDBR enzymatic activity with pulegone.; Figure S13: GC-MS analysis of CsDBR enzymatic activity with α-methylcinnamaldehyde.; Table S1: Quality summary of sample sequencing data.; Table S2: Length distribution of transcripts and unigenes.; Table S3: Summary statistics of transcriptome assembly.; Table S4: Functional annotation statistics of *C. sinense* unigenes in public databases.; Table S5: Primers used in this study.

## Abbreviations

The following abbreviations are used in this manuscript:

CDS	Coding sequence
DEGs	Differentially expressed genes
GC-MS	Gas chromatography-mass spectrometry
GO	Gene Ontology
HS-SPME	Headspace solid-phase microextraction
HS-SPME-GC-MS	Headspace solid-phase microextraction coupled with gas chromatography-mass spectrometry
IPTG	Isopropyl β-D-1-thiogalactopyranoside
KEGG	Kyoto Encyclopedia of Genes and Genomes
KOG	EuKaryotic Orthologous Groups
MeJA	Methyl jasmonate
NR	Non-redundant protein
NT	Non-redundant nucleotide
ORF	Open reading frame
Pfam	Protein families database
qRT-PCR	Quantitative reverse transcription polymerase chain reaction
SDS-PAGE	Sodium dodecyl sulfate-polyacrylamide gel electrophoresis
TFs	Transcription factors
VOCs	Volatile organic compounds
MVA	Mevalonic acid
MVAP	Mevalonic acid 5-phosphate
MVAPP	5-Diphosphomevalonic acid
IPP	Isopentenyl diphosphate
FPP	Farnesyl diphosphate
DXP	1-deoxy-D-xylulose 5-phosphate
MEPP	2-C-Methyl-D-erythritol 4-phosphate
MCP-ME	4-(Cytidine 5′-diphospho)-2-C-methyl-D-erythritol
CDP-MEP	2-Phospho-4-(cytidine 5′-diphospho)-2-C-methyl-D-erythritol
MECP	2-C-Methyl-D-erythritol 2,4-cyclodiphosphate
HMBPP	1-Hydroxy-2-methyl-2-butenyl 4-diphosphate
DMAPP	Dimethylallyl diphosphate
GPP	Geranyl pyrophosphate
GGPP	Geranylgeranyl diphosphate
AACT	Acetyl-CoA acetyltransferase
HMGS	Hydroxymethylglutaryl-CoA
HMGR	Hydroxymethylglutaryl-CoA reductase synthase
MVK	Mevalonate kinase
PMK	Phosphomevalonate kinase
MVD	Mevalonate diphosphate decarboxylase
FPPS	Farnesyl diphosphate synthase
DXS	1-deoxy-D-xylulose-5-phosphate synthase
DXR	1-deoxy-D-xylulose-5-phosphate reductase
MCT	2-C-methyl-D-erythritol 4-phosphate cytidylyltransferase
CMK	4-diphosphocytidyl-2-C-methyl-D-erythritol kinase
MECS	2-C-methylerythritol 2,4-cyclodiphosphate synthase
HDS	4-hydroxy-3-methylbut-2-en-1-yl diphosphate synthase
HDR	4-hydroxy-3-methylbut-2-enyl diphosphate reductase
IDI	Isopentenyl pyrophosphate isomerase
GPPS	Geranyl pyrophosphate synthase
GGPPS	Geranylgeranyl diphosphate synthase
PSY	Phytoene synthase
PDS	Phytoene desaturase
Z-ISO	15-cis-ζ-carotene isomerase
ZDS	ζ-carotene desaturase
CRTISO	Carotenoid isomerase
LCY-E	Lycopene ε-cyclase
LCY-B	Lycopene β-cyclase
CHY-E	ɛ-hydroxylase
CHY-B	β-hydroxylase
VDE	Violaxanthin de-epoxidase
ZEP	Zeaxanthin epoxidase
NXS	Neoxanthin synthase
NCED	Nine-cis-epoxycarotenoid dioxygenase
CCD	Carotenoid cleavage dioxygenase
