# Supplementary material for: Functional Characterization of Double-Bond Reductases in Dihydro-β-Ionone Biosynthesis in Cymbidium sinense
Source: Plants (Basel). 2025 Dec 13;14(24):3804. doi: 10.3390/plants14243804 (PMC12736756; doi:10.3390/plants14243804)
Supplement: Supplementary file 1 [file plants-14-03804-s001.zip › plants-3995850-supplementary.pdf]

Supplementary Materials

Supplementary Figures

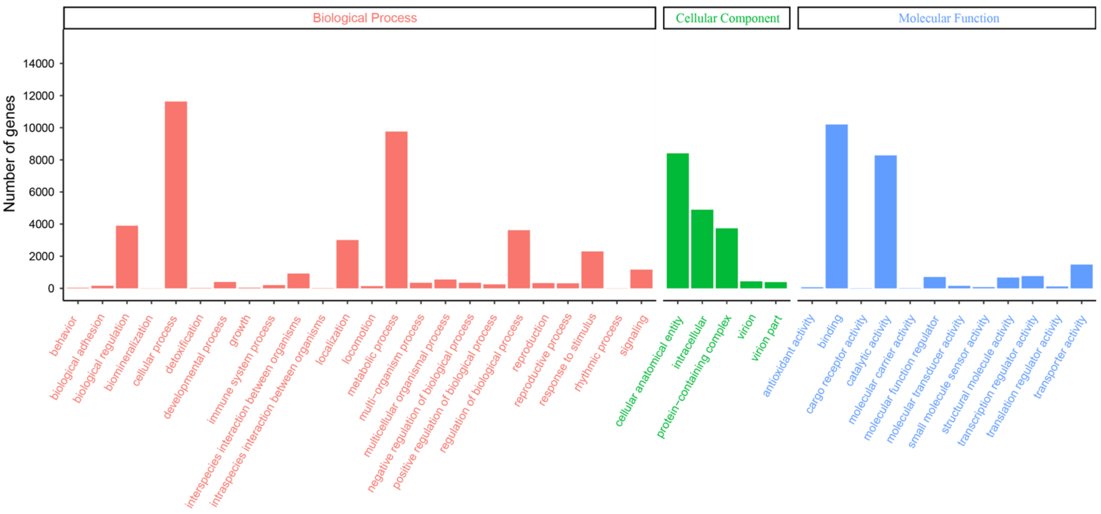

Figure S1. Gene Ontology (GO) classification of annotated unigenes from *C. sinense* transcriptome.

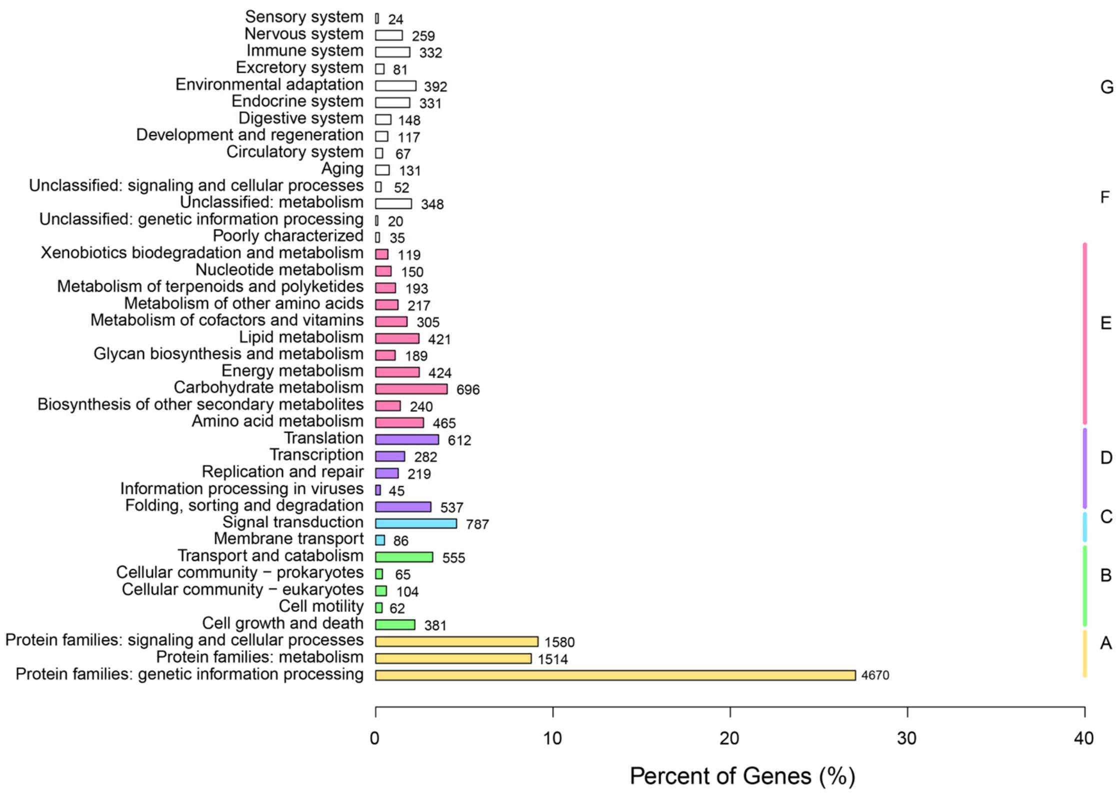

Figure S2. KEGG pathway classification of annotated unigenes from *C. sinense* transcriptome.

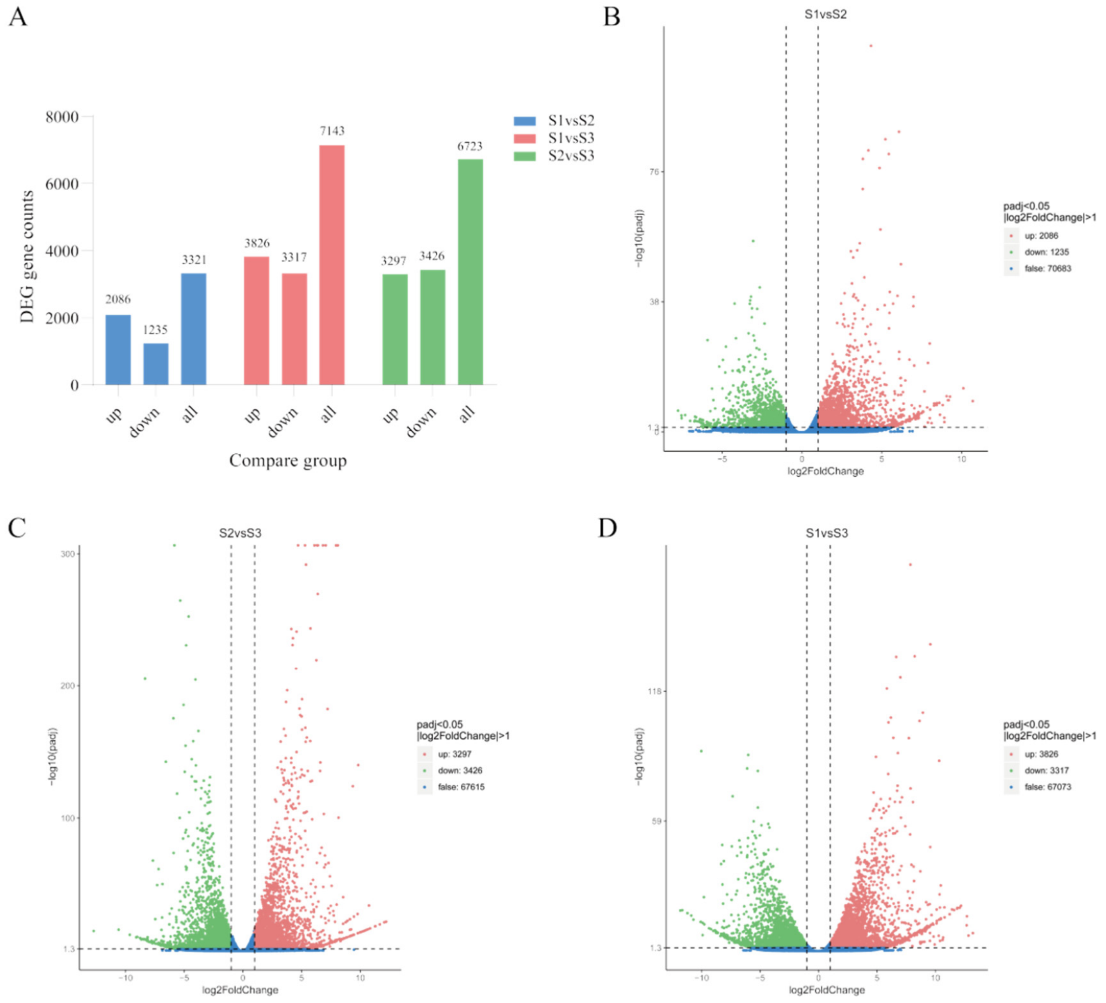

Figure S3. Analysis of differentially expressed genes (DEGs) among three comparison groups. (A) Histogram of the number of differential genes. (B) Differential gene Volcano plot of S1 vs. S2. (C) Differential gene Volcano plot of S2 vs. S3. (D) Differential gene Volcano plot of S1 vs. S3.

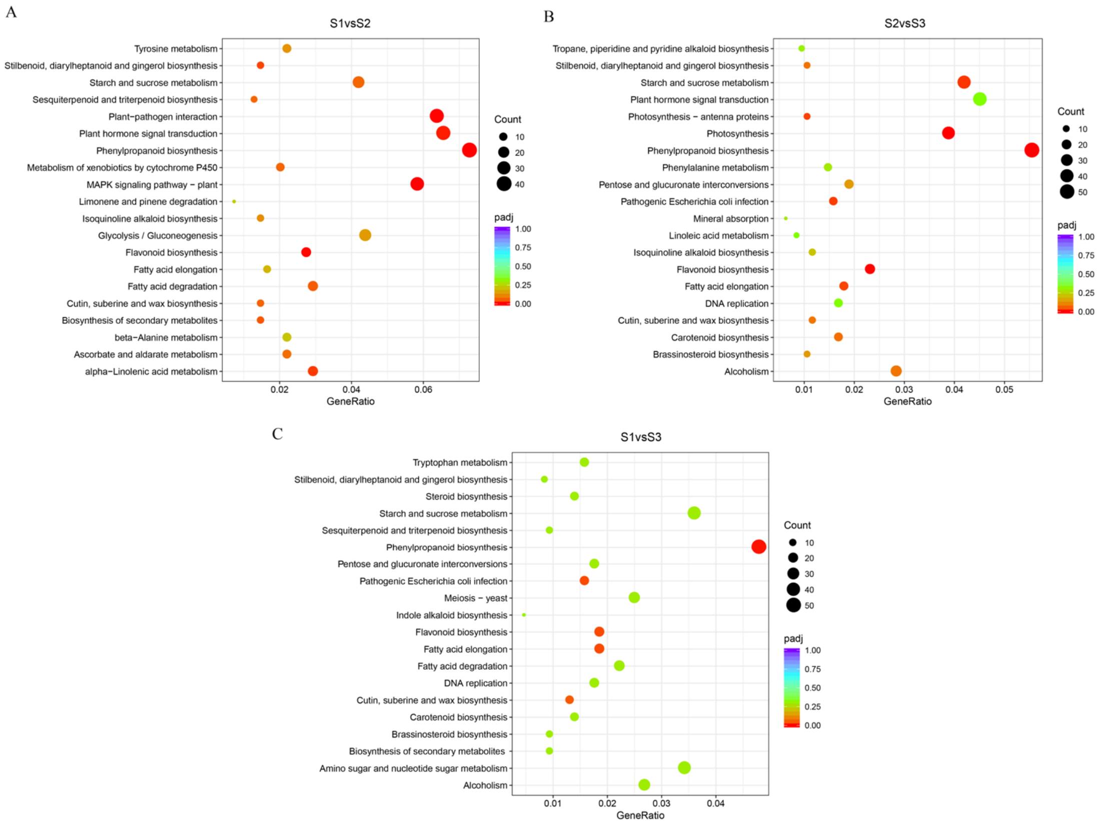

Figure S4. KEGG pathway enrichment analysis of differentially expressed genes (DEGs).

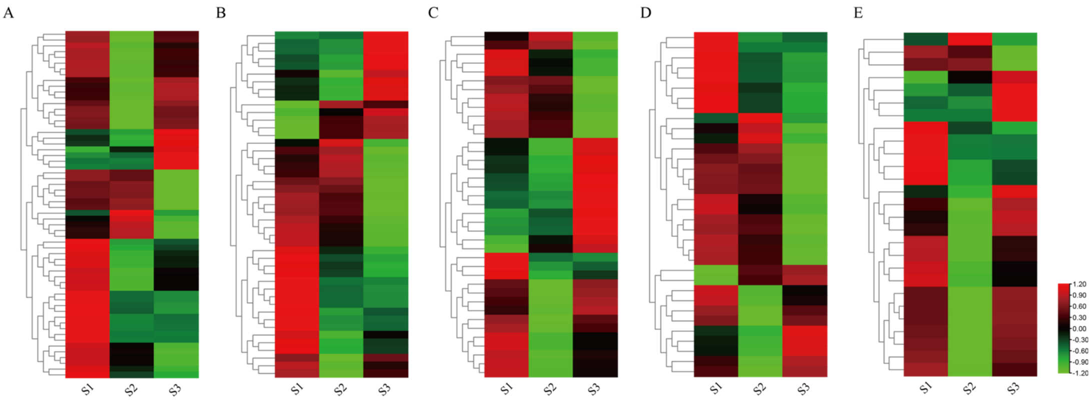

Figure S5. Gene expression of transcription factors. (A) AP2/ERF, (B) MYB, (C) C2H2, (D) bHLH, (E) WRKY.

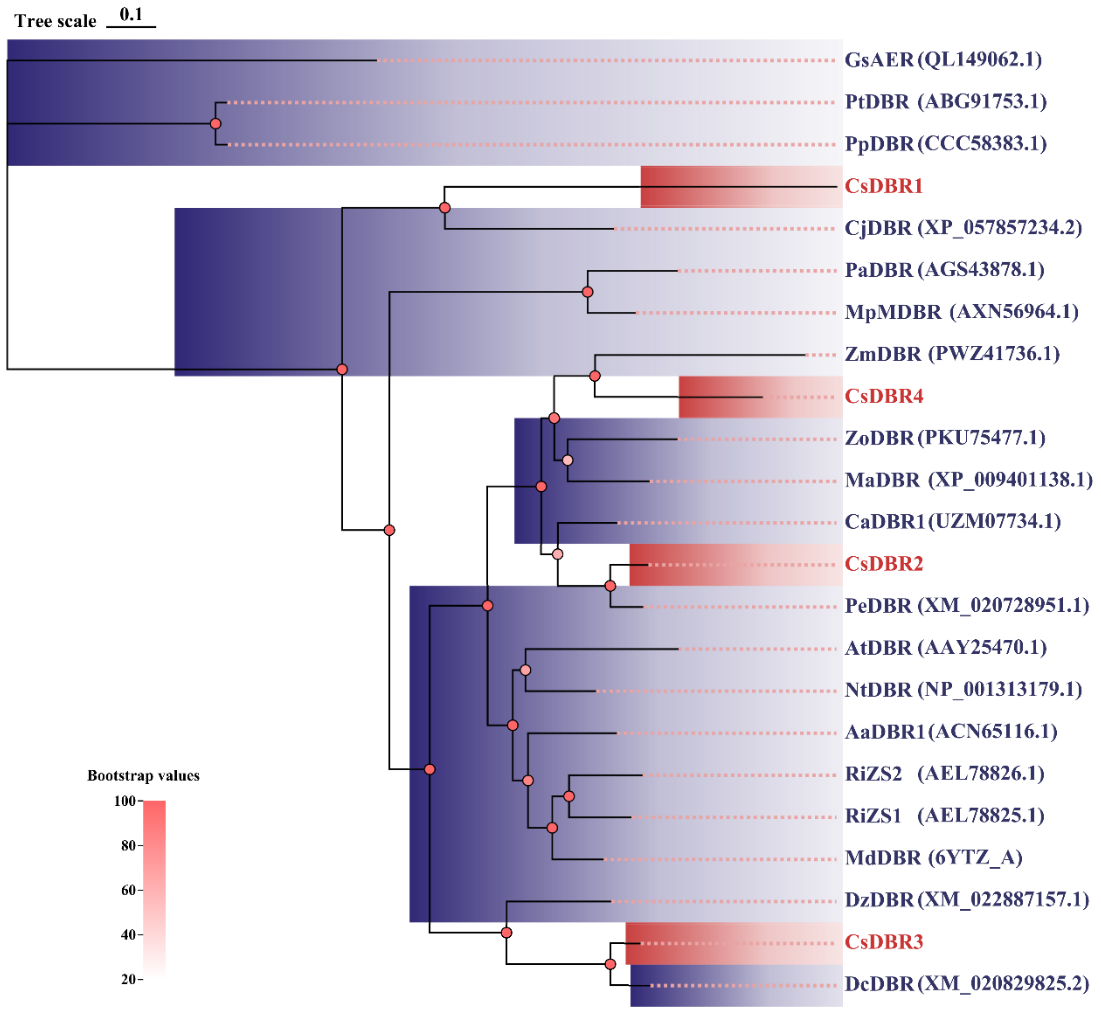

Figure S6. Comparative phylogenetic analysis of CsDBRs and other plant DBR proteins. Phylogenetic trees were constructed by the maximum likelihood method using TBtools and visualized with ChiPlot. Sequences analyzed in this study are highlighted in red font. Species and accession numbers: *Gloriosa superba* GsAER (QLI49062.1); *Pinus taeda* PtDBR (ABG91753.1); *Pinus pinaster* PpDBR (CCC58383.1); *Cryptomeria japonica* CjDBR (XP_057857234.2); *Plagiochasma appendiculatum* PaDBR (AGS43878.1); *Marchantia paleacea* MpMDBR (AXN56964.1); *Zea mays* ZmDBR (PWZ41736.1); *Zingiber officinale* ZoDBR (PKU75477.1); *Musa acuminata* MaDBR (XP_009401138.1); *Colchicum autumnale* CaDBR1 (UZM07734.1); *Phalaenopsis equestris* PeDBR (XM_020728951.1); *Arabidopsis thaliana* AtDBR (AAY25470.1); *Nicotiana tabacum* NtDBR (NP_001313179.1); *Artemisia annua* AaDBR1 (ACN65116.1); *Rubus idaeus* RiZS2 (AEL78826.1); *Rubus idaeus* RiZS1 (AEL78825.1); *Malus domestica* MdDBR (6YTZ_A); *Durio zibethinus* DzDBR (XM_022887157.1); *Dendrobium catenatum* DcDBR (XM_020829825.2).

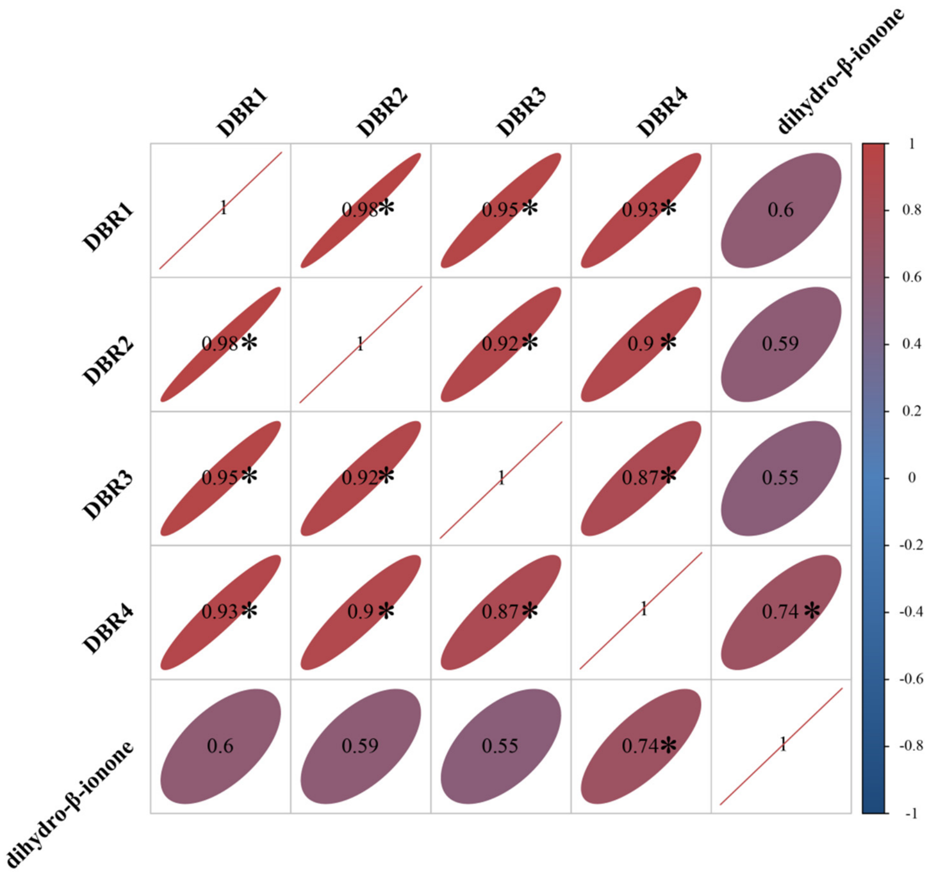

Figure S7. Heat map representation of correlation analysis between dihydro-β-ionone and selected *DBR* genes. Statistical analysis was performed by Pearson. "Asterisk" denotes significant differences (p<0.05). Color card and Number indicate correlation coefficient.

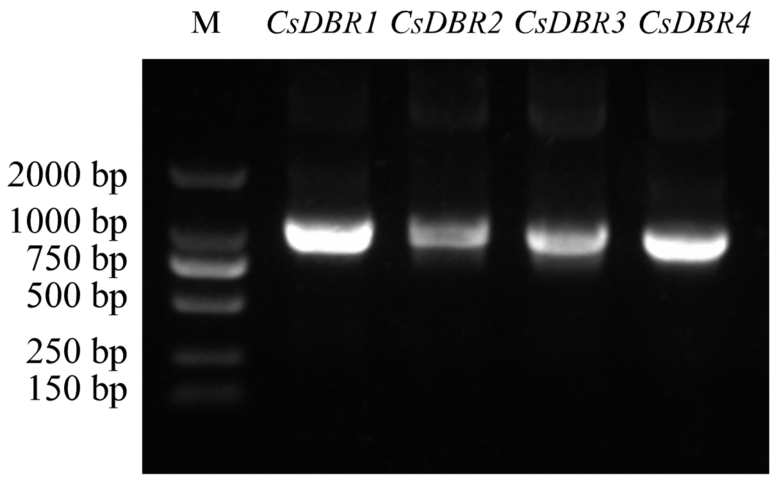

Figure S8. Agarose gel electrophoresis of *CsDBR* amplification products. M: D2000 DNA marker.

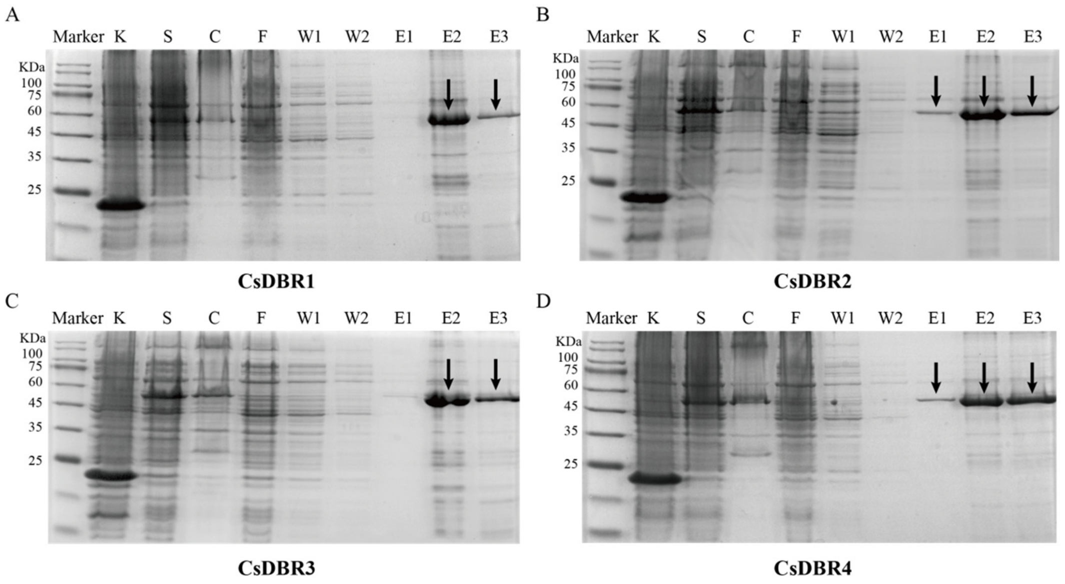

Figure S9. SDS-PAGE of the purified CsDBR enzyme. Lane K: Supernatant protein from the pET-32a empty vector. Lane S: Soluble supernatant fraction of the CsDBR protein. Lane C: Insoluble precipitate fraction of the CsDBR protein. Lane F: Flow-through fraction from the CsDBR recombinant protein purification. Lane W1 and W2: Wash fractions from the CsDBR recombinant protein purification. Lane E1, E2, and E3: Elution fractions of the purified CsDBR recombinant protein.

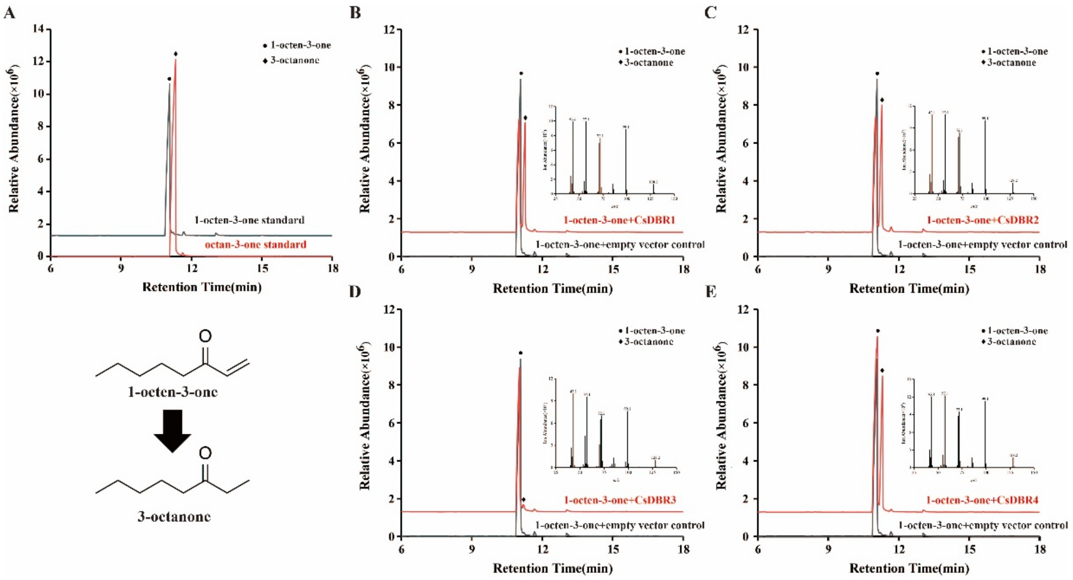

Figure S10. GC-MS analysis of CsDBR enzymatic activity with 1-octen-3-one. Chromatograms for (A) authentic standards of 1-octen-3-one and 3-octanone, and assays with (B) CsDBR1, (C) CsDBR2, (D) CsDBR3, and (E) CsDBR4. Circle and rhombus represent 1-octen-3-one and 3-octanone.

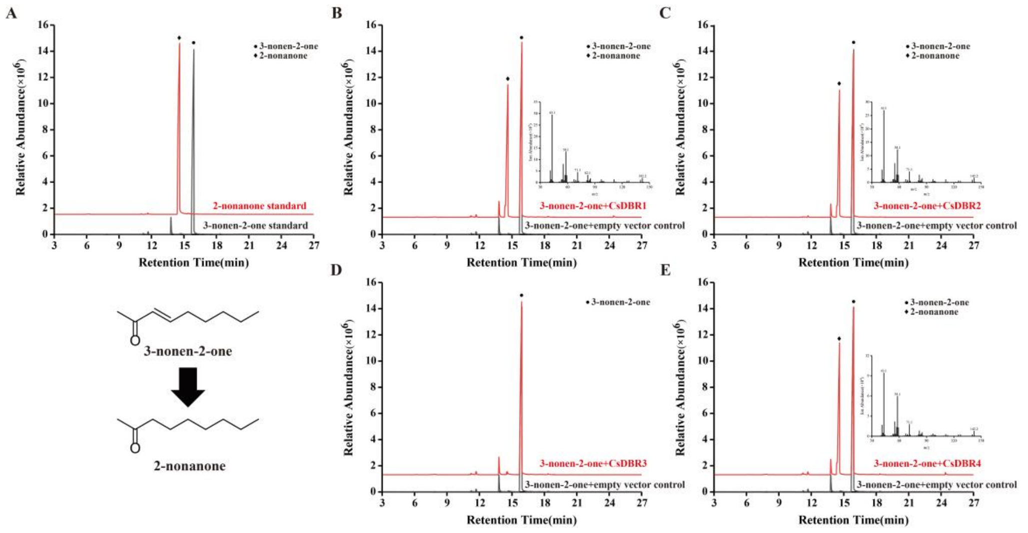

Figure S11. GC-MS analysis of CsDBR enzymatic activity with 3-nonen-2-one. Chromatograms for (A) authentic standards of 3-nonen-2-one and 2-nonanone, and assays with (B) CsDBR1, (C) CsDBR2, (D) CsDBR3, and (E) CsDBR4. Circle and rhombus represent 3-nonen-2-one and 2-nonanone.

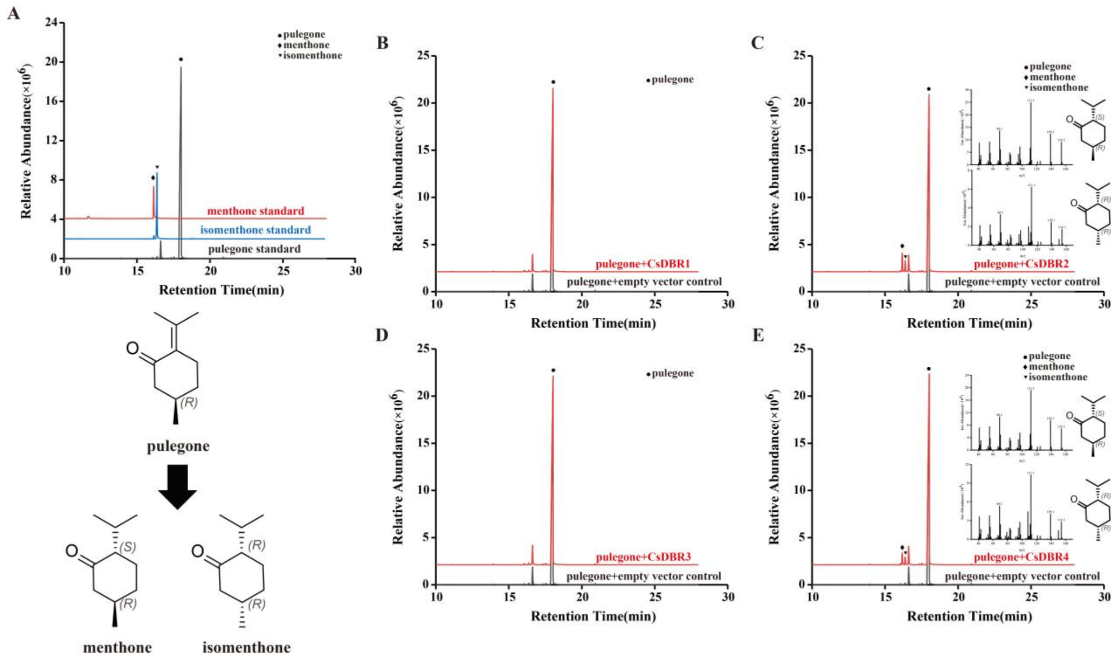

Figure S12. GC-MS analysis of CsDBR enzymatic activity with pulegone. Chromatograms for (A) authentic standards of pulegone, menthone and isomenthone, and assays with (B) CsDBR1, (C) CsDBR2, (D) CsDBR3, and (E) CsDBR4. Circle, rhombus and triangle represent pulegone, menthone and isomenthone.

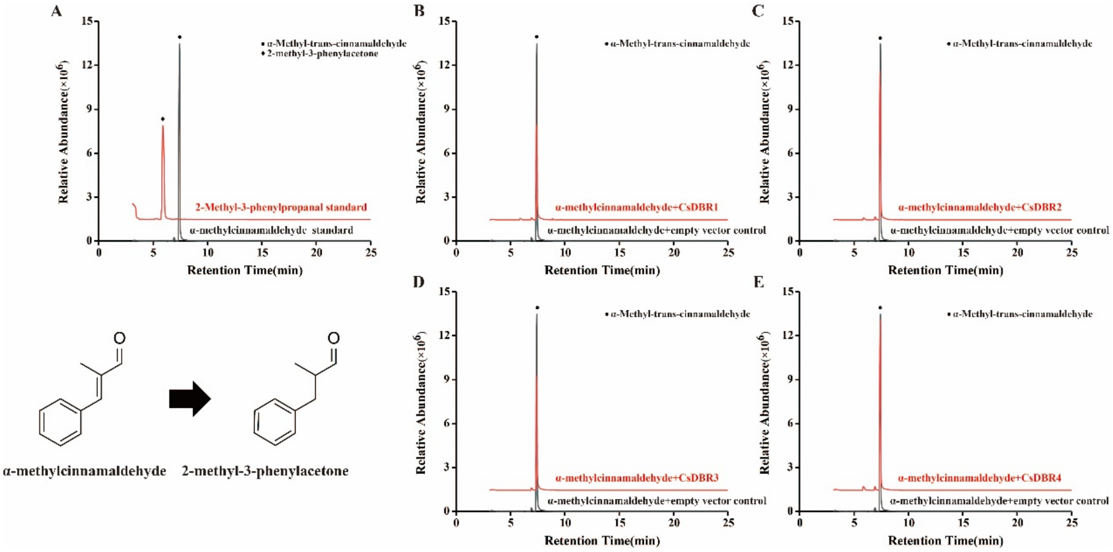

Figure S13. GC-MS analysis of CsDBR enzymatic activity with α-methylcinnamaldehyde. Chromatograms for (A) authentic standards of α-methylcinnamaldehyde and 2-methyl-3-phenylacetone, and assays with (B) CsDBR1, (C) CsDBR2, (D) CsDBR3, and (E) CsDBR4. Circle and rhombus represent α-methylcinnamaldehyde and 2-methyl-3-phenylacetone.

Supplementary Tables

Table S1. Quality summary of sample sequencing data.

Sample	Raw date(GB)	Clean date(GB)	Q20%	Q30%	error rate	GC pct
S1_1	8.14	7.96	97.88	93.78	0.03	46.76
S1_2	7.03	6.93	98.45	95.05	0.02	47.57
S1_3	6.87	6.73	98.05	94.25	0.02	46.72
S2_1	7.14	7	98.01	94.06	0.03	46.07
S2_2	6.84	6.64	97.81	93.85	0.03	46.29
S2_3	6.83	6.63	97.94	94.19	0.03	46.06
S3_1	6.81	6.68	97.94	93.91	0.03	43.89
S3_2	6.93	6.86	98.07	93.98	0.03	44.98
S3_3	6.89	6.71	98.02	94.1	0.03	44.47

Table S2. Length distribution of transcripts and unigenes.

Transcript length interval	300-500bp	500-1kbp	1k-2kbp	>2kbp	Total
Number of Transcripts	55,158	44,906	37,858	39,249	177,171
Number of Unigenes	28,006	24,292	13,059	11,346	76,703

Table S3. Summary statistics of transcriptome assembly.

Type	min length	mean length	median length	max length	N50	N90	total nucleotides
Transcript	301	1,378	815	16,758	2,276	539	244,098,795
Unigene	301	1,112	628	16,758	1,782	454	85,301,823

Table S4. Functional annotation statistics of *C. sinense* unigenes in public databases.

Database	Number of Unigenes	Percentage (%)
Annotated in NR	30,489	39.74
Annotated in NT	25,593	33.36
Annotated in KO	9,083	11.84
Annotated in SwissProt	18,218	23.75
Annotated in PFAM	19,726	25.71
Annotated in GO	19,723	25.71
Annotated in KOG	5,505	7.17
Annotated in all Databases	3,460	4.51
Annotated in at least one Database	38,446	50.12

Table S5. Primers used in this study.

Primer name	Sequence (5′–3′)
CsDXS-qRT-F	TGCATGAACTGGCAGCCA
CsDXS-qRT-R	TGTCCATCAACAGGCCCG
CsDXR-qRT-F	CCGGCGGAAATTGGAGGT
CsDXR-qRT-R	CCAACCCACCGAGTTCCC
CsGGPPS-qRT-F	TGCGCGATCCATTGGGTT
CsGGPPS-qRT-R	TCTCCAACCCCACCAGCT
CsPSY-qRT-F	CAGGCGGCTCTTGTGGAG
CsPSY-qRT-R	GCATACTCCGCGCAGACT
CsZ-ISO-qRT-F	GCTTGATCGGCCACCACT
CsZ-ISO-qRT-R	TTGCCGCAAAGGGAACGA
CsZDS-qRT-F	CGCGCTTGCTCTAGCTCT
CsZDS-qRT-R	TGTATGCTTGCGCGGGTT
CsCRTISO-qRT-F	TGGGTTCGTTCTCCGTGC
CsCRTISO-qRT-R	CCTTCCCTGCTCCGCTTC
CsLCYE-qRT-F	GCTGATGTCCCGCTTGGA

Table S5. (Continued)

Primer name	Sequence (5′–3′)
CsLCYE-qRT-R	AGCACCGAACGCAAGGTT
CsVDE-qRT-F	TGGTGCAGTTGACAGCCT
CsVDE-qRT-R	TGTTTGCTGCGCATGCTG
CsDBR1-qRT-F	TGCTTGGGCATTGGGGAG
CsDBR1-qRT-R	GTAAGGTGCCGGCCATGT
CsDBR2-qRT-F	GCGCAATGCTCGATGCTG
CsDBR2-qRT-R	GGGGACGACGAACAGCAA
CsDBR3-qRT-F	GATTAGGGTTCCGGCGGG
CsDBR3-qRT-R	CCGGGCTGAAATGGAGGG
CsDBR4-qRT-F	CAGAGGGTTCGCGGTGTT
CsDBR4-qRT-R	CGTTCTCAAGGCCCTCCG
CsACTIN-qRT-F	CTGGGATGGTGAAGGCCG
CsACTIN-qRT-R	TCTGTCCCATCCCGACCA
CsDBR1-F	GATGAAGGTGGAGAACAGATATG
CsDBR1-R	TTAGATTTCACTCACCTGCAC
CsDBR2-F	GAATGACGGAGAACCGAGAG
CsDBR2-R	TCATTCCCAAAATATGAACTTTCCC
CsDBR3-F	GAAACAATGGAGGCGGCGAAGA
CsDBR3-R	TCAATCCCTTGAAATACAAACAACCTGC
CsDBR4-F	GATGGCAGGGAAGAAGGTTG
CsDBR4-R	CTATTCCCTATCCACCGCCA
pET32a-CsDBR1-F	gccatggctgatatcggatccATGAAGGTGGAGAACAGATATGTTACA
pET32a-CsDBR1-R	ctcgagtgcggccgcaagcttGATTTCACTCACCTGCACTAATTTCT
pET32a-CsDBR2-F	gccatggctgatatcggatccATGACGGAGAACCGAGAGGAG
pET32a-CsDBR2-R	ctcgagtgcggccgcaagcttTTCCCAAAATATGAACTTTCCCA
pET32a-CsDBR3-F	gccatggctgatatcggatccATGGAGGCGGCGAAGACG
pET32a-CsDBR3-R	ctcgagtgcggccgcaagcttATCCCTTGAAATACAAACAACCTGC

Table S5. (Continued)

Primer name	Sequence (5′–3′)
pET32a-CsDBR4-F	gccatggctgatatcggatccATGGCAGGGAAGAAGGTTGTT
pET32a-CsDBR4-R	ctcgagtgcggccgcaagcttTTCCCTATCCACCGCCACC
pGreenII 62-SK-CsDBR1-F	cgctctagaactagtggatccATGAAGGTGGAGAACAGATATGTTACA
pGreenII 62-SK-CsDBR1-R	gtcgacggtatcgataagcttGATTTCACTCACCTGCACTAATTTCT
pGreenII 62-SK-CsDBR2-F	cgctctagaactagtggatccATGACGGAGAACCGAGAGGAG
pGreenII 62-SK-CsDBR2-R	gtcgacggtatcgataagcttTTCCCAAAATATGAACTTTCCCA
pGreenII 62-SK-CsDBR4-F	cgctctagaactagtggatccATGGCAGGGAAGAAGGTTGTT
pGreenII 62-SK-CsDBR4-R	gtcgacggtatcgataagcttTTCCCTATCCACCGCCACC
p35S-GFP-CsDBR1-F	ttacaattacagtcgactagtgATGTCGACAGAGAAGGTCGGTAA
p35S-GFP-CsDBR1-R	cctcgcccttgctcaccatggaTTCGTGAGCAATCAAGACGACC
p35S-GFP-CsDBR2-F	ttacaattacagtcgactagtgATGACGGAGAACCGAGAGGAG
p35S-GFP-CsDBR2-R	cctcgcccttgctcaccatggaTTCCCAAAATATGAACTTTCCCA
p35S-GFP-CsDBR3-F	ttacaattacagtcgactagtgATGGAGGCGGCGAAGACG
p35S-GFP-CsDBR3-R	cctcgcccttgctcaccatggaATCCCTTGAAATACAAACAACCTGC
p35S-GFP-CsDBR4-F	ttacaattacagtcgactagtgATGGCAGGGAAGAAGGTTGTT
